# Practical and Efficient Synthesis of α-Aminophosphonic Acids Containing 1,2,3,4-Tetrahydroquinoline or 1,2,3,4-Tetrahydroisoquinoline Heterocycles

**DOI:** 10.3390/molecules21091140

**Published:** 2016-08-31

**Authors:** Mario Ordóñez, Alicia Arizpe, Fracisco J. Sayago, Ana I. Jiménez, Carlos Cativiela

**Affiliations:** 1 Centro de Investigaciones Químicas-IICBA, Universidad Autónoma del Estado de Morelos, Av. Universidad 1001, Cuernavaca 62209, Morelos, Mexico; 2 Departamento de Química Orgánica, Universidad de Zaragoza-CSIC, ISQCH, Zaragoza 50009, Spain; aliceiw@gmail.com (A.A.); jsayago@unizar.es (F.J.S.); anisjim@unizar.es (A.I.J.)

**Keywords:** α-aminophosphonic acids, *N*-acyliminium ions, conformationally constrained

## Abstract

We report here a practical and efficient synthesis of α-aminophosphonic acid incorporated into 1,2,3,4-tetrahydroquinoline and 1,2,3,4-tetrahydroisoquinoline heterocycles, which could be considered to be conformationally constrained analogues of pipecolic acid. The principal contribution of this synthesis is the introduction of the phosphonate group in the *N*-acyliminium ion intermediates, obtained from activation of the quinoline and isoquinoline heterocycles or from the appropriate δ-lactam with benzyl chloroformate. Finally, the hydrolysis of phosphonate moiety with simultaneous cleavage of the carbamate afforded the target compounds.

## 1. Introduction

There is a continuously growing interest in the development of new peptidomimetics, compounds that mimic the bioactive conformation and action of therapeutic peptides while possessing greater bioavailability and stability and less undesirable effects. In this regard, the incorporation of rigid unusual secondary α-amino acids, where the nitrogen is involved in a ring, may result in significant consequences for the conformation of peptidomimetics as synthetic tools for drug discovery [1,2]. Some of the most important molecules are the 1,2,3,4-tetrahydroquinoline-2-carboxylic acid **1** [3,4] and 1,2,3,4-tetrahydroisoquinoline-1-carboxylic acid **2** [5,6], which are conformationally constrained analogues of unnatural pipecolic acid, and 1,2,3,4-tetrahydroisoquinoline-3-carboxylic acid (Tic) **3** [7,8], which is considered a conformationally constrained analogue of phenylalanine (Phe). These compounds are important unnatural α-amino acids, and they are used as key intermediates in organic synthesis for the preparation of biologically active compounds. Therefore, much effort has been dedicated to the preparation of these compounds.

**Figure 1:**
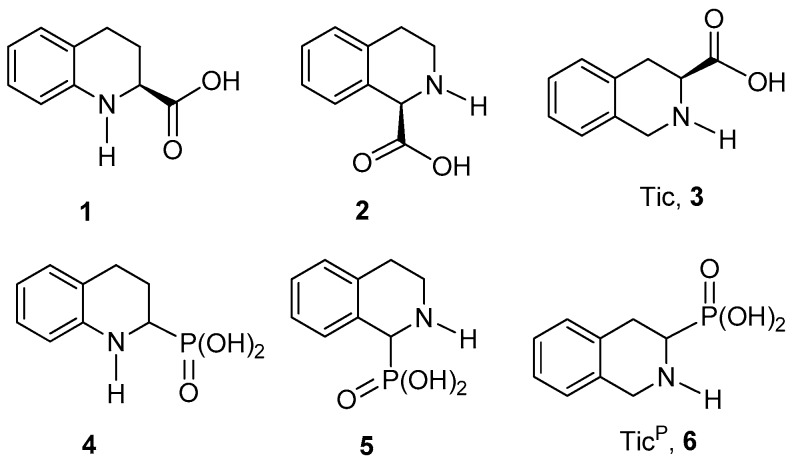
α-Amino acids characterized by a tetrahydroquinoline or tetrahydroisoquinoline heterocyles and their α-amino phosphonic analogues.

On the other hand, the α-aminoalkylphosphonic acids are probably the most important analogues of the α-amino acids, obtained by isosteric substitution of the planar and less bulky carboxylic acid (CO_2_H) by a tetrahedral phosphonic acid functionality (PO_3_H_2_). This class of compounds is currently attracting interest in organic and medicinal chemistry, due to their important biological and pharmacological properties [9,10,11,12]. The great importance of this type of compound has prompted organic chemists to report numerous procedures for their racemic or stereoselective synthesis [13,14,15,16,17,18], principally using the diastereoselective and enantioselective Pudovik [19,20] and Kabachnik–Fields [21,22,23,24,25] reactions for acyclic α-aminoalkylphosphonates, and through *N*-acyl iminium ions for the synthesis of cyclic derivatives [26,27,28,29,30,31,32,33,34]. However, to the best of our knowledge, the synthesis of 1,2,3,4-tetrahydroquinoline-2-phosphonic acid **4** and 1,2,3,4-tetrahydroisoquinoline-1-phosphonic acid **5** analogues [35,36,37,38] has not yet been described in the literature, whereas the synthesis of 1,2,3,4-tetrahydroisoquinoline-3-phosphonic acid **6** has been recently described by our research group [39,40] (Figure 1).

Considering the high value of these non-coded compounds in connection with our current research interest in the synthesis of novel conformationally restricted α-aminophosphonic acids [26,27,28,41,42,43], we now report herein the practical and efficient synthesis of α-aminophosphonic acids incorporating 1,2,3,4-tetrahydroquinoline **4**, and 1,2,3,4-tetrahydroisoquinoline rings **5** and **6**, which could be considered to be conformationally constrained analogues of pipecolic acid. The principal contribution of this work is the regioselective introduction of the phosphonate group in the *N*-acyliminium ions derivatives obtained from activation of the quinoline and isoquinoline nucleous or from the appropriate δ-lactams with benzyl chloroformate.

## 2. Results and Discussion

**Scheme 1:**
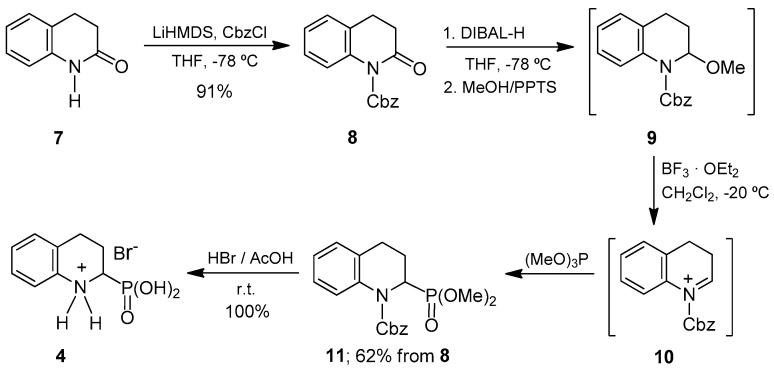
Synthesis of 1,2,3,4-tetrahydroquinoline-2-phosphonic acid **4** from lactam **7**.

For the synthesis of 1,2,3,4-tetrahydroquinoline-2-phosphonic acid **4**, we proposed the *N*-acyliminium ions as suitable precursors, considering that the well-known reduction of the carbonyl group of lactams such as the 3,4-dihydro-2(1*H*)-quinolinone **7**, and their subsequent transformation into a *N*-acyliminium ion, is one of the methods that allows the incorporation of the phosphonate functionality into the α position of the nitrogen atom such as we have previously described [26,27,28]. These proposals prompted us to explore further application of the *N*-acyliminium strategy for the synthesis of the target compound. In this context, the commercially available 3,4-dihydro-2(1*H*)-quinolinone **7** was reacted with lithium bis(trimethylsilyl)amide (LiHMDS) as a base and benzyl chloroformate in THF at −78 °C, to obtain the *N*-Cbz-3,4-dihydro-2(1*H*)-quinolinone **8** in 91% yield. Reduction of the carbonyl group in **8** with diisobutylaluminium hydride (DIBAL-H) and subsequent reaction with methanol and catalytic amounts of pyridinium *p*-toluenesulfonate (PPTS), gave the methoxyaminal **9**, which was treated immediately with trimethyl phosphite in the presence of BF_3_·OEt_2_, obtaining the dimethyl *N*-Cbz-1,2,3,4-tetrahydroquinoline-2-phosphonate **11** in 62% via the *N*-acyliminium ion **10** in 62% yield from **8**. Treatment of this *N*-Cbz-protected phosphonate with a 33% solution of hydrogen bromide in acetic acid, afforded the 1,2,3,4-tetrahydroquinoline-2-phosphonic acid **4** as hydrobromide in quantitative yield (Scheme 1).

The literature has described the hydrophosphonylation of quinoline derivatives to obtain the 1,2-dihydroquinolin-2-ylphosphonate and 2,4-diphosphono-1,2,3,4-tetrahydroquinoline derivatives using activating agents [44,45,46,47,48]. These reactions proceed via quinolinium ions that can be viewed as counterparts of the above-mentioned iminium species. Thus, the addition of an acyl chloride to quinoline can be considered as a way to generate the iminium ion necessary for subsequent regioselective incorporation of the phosphonate functionality α to the nitrogen atom.

**Scheme 2:**
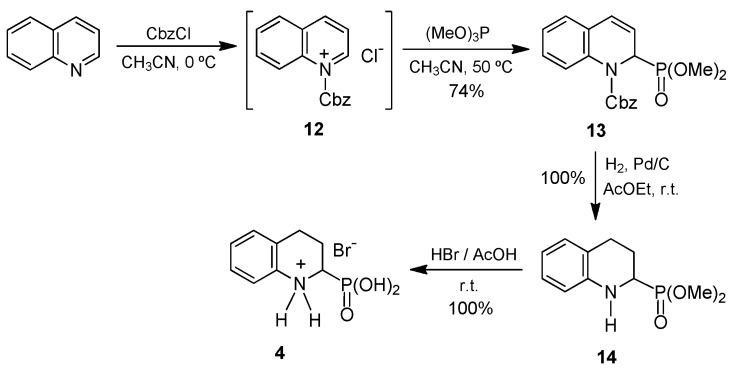
Synthesis of 1,2,3,4-tetrahydroquinoline-2-phosphonic acid **4** from quinoline.

Based on this precedent, we decided to compare the efficiency of the *N*-acylquinolinium salts as intermediates in the synthesis of α-aminophosphonic acid **4**. For this purpose, benzyl chloroformate was added to quinoline and the *N*-Cbz-quinolinium chloride formed **12** was reacted with trimethyl phosphite in acetonitrile at 50 °C, obtaining the dimethyl *N*-Cbz-1,2-dihydroquinoline-2-phosphonate derivative **13** in 74% yield from quinolone. The diphosphonylation products at the 2- and 4-positions of the quinoline ring were not detected. The regioselectivity observed in the addition of trimethyl phosphite to the *N*-acylquinolinium ion **12** may be attributed to the electron-withdrawing character of the benzyloxycarbonyl group, which increases the electrophilic nature of the carbon adjacent to the nitrogen atom. Additionally, the benzyloxycarbonyl group was selected for its easy incorporation and removal under mild conditions. Thus, the catalytic hydrogenation resulted in simultaneous removal of the Cbz group and reduction of the double bond at positions 3,4 to afford the α-aminophosphonate **14**, which, by treatment with hydrogen bromide in acetic acid, gave the 1,2,3,4-tetrahydroquinoline-2-phosphonic acid **4** as hydrobromide. The latter two steps proceeded quantitatively and obviated the need for purification (Scheme 2).

The 1,2,3,4-tetrahydroquinoline-2-phosphonic acid **4** was obtained in 74% overall yield from quinoline following the synthetic strategy in Scheme 2, which compares favorably with the 56% global yield achieved in Scheme 1, when starting from quinolinone **1**. Accordingly, the superior global yield, together with the much lower price of quinoline in comparison with 3,4-dihydro-2(1*H*)-quinolinone **7**, makes the methodology in Scheme 2 more advantageous than the lactam-based one for the preparation of α-aminophosphonic acid **4**. From an operational viewpoint, both routes required few purification steps—by column chromatography—of intermediate compounds.

We next addressed the preparation of 1,2,3,4-tetrahydroisoquinoline-1-phosphonic acid **5**. Similarly to that described above for the quinoline counterpart, the preparation of *N*-substituted-1,2-dihydroisoquinoline-1-phosphonates through the isoquinolinium salts formed by reaction of isoquinoline with acyl chlorides and subsequent addition of trialkyl phosphites has been described [49,50]. Thus, the synthesis of dimethyl *N*-Cbz-1,2-dihydroisoquinoline-1-phosphonate should be straightforward, as shown above for the preparation of dimethyl *N*-Cbz-1,2-dihydroquinoline-2-phosphonate **13**.

On the other hand, the lactam to be used as a starting product for the preparation of the target amino acid **5** is not easily available from commercial sources in this case. This fact together with the advantageous preparation of amino acid **4** via the formation of quinolinium salt prompted us to undertake the synthesis of 1,2,3,4-tetrahydroisoquinoline-1-phosphonic acid following an analogous synthetic route.

**Scheme 3:**
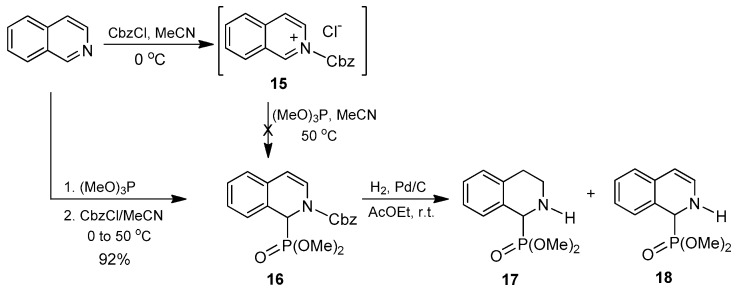
First attempt to synthetize 1,2,3,4-tetrahydroisoquinoline-1-phosphonic acids derivatives.

Thus, using the reaction conditions described above, the isoquinoline was reacted with benzyl chloroformate followed by the addition of trimethyl phosphite in acetonitrile at 50 °C. However, under these reaction conditions, the desired compound **16** was not obtained. It is noteworthy that during the addition of benzyl chloroformate to isoquinoline, a yellow solid was formed, which was later identified as the isoquinolinium salt **15**. This compound remained unaltered upon addition of trimethyl phosphite so that the formation of the expected 1,2-dihydroisoquinoline-1-phosphonate **16** did not take place. We reasoned that if the trimethyl phosphite was present in the reaction medium before benzyl chloroformate was added, the isoquinolinium ion formed **16** would immediately react with it, thus preventing precipitation. To our delight, when the order of addition of these reagents was exchanged (that is, trimethyl phosphite prior to benzyl chloroformate), the dimethyl *N*-Cbz-1,2-dihydroisoquinoline-1-phosphonate **16** was obtained at 92% yield. In the next step, we carried out the catalytic hydrogenation of the double bond in **16** under an atmospheric pressure of hydrogen gas and using Pd/C as a catalyst, to generate the tetrahydroisoquinoline moiety. However, the Cbz group in **16** was readily eliminated at room temperature, whereas the double bond at positions 3,4 was only partially hydrogenated even after long reaction times. As a consequence, mixtures of the desired tetrahydroisoquinoline derivative **17** and the analogous dihydroisoquinoline **18** were obtained. Attempts to improve this result by changing the solvent as well as by increasing the reaction temperature or hydrogen gas pressure proved unsuccessful and a mixture of **17** and **18** was always obtained (Scheme 3).

**Scheme 4:**
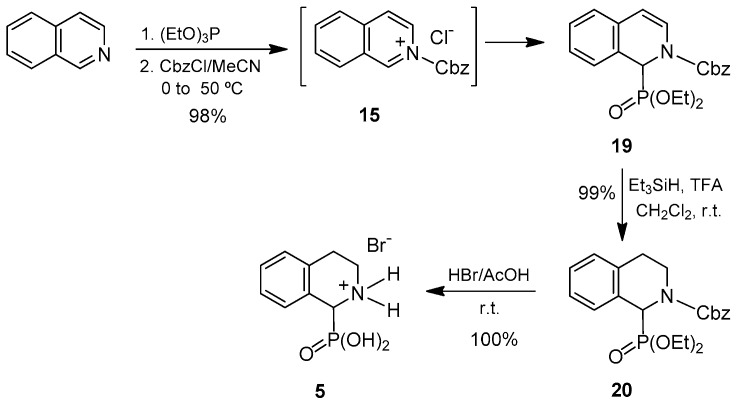
Synthesis of 1,2,3,4-tetrahydroisoquinoline-1-phosphonic acid **5** from isoquinoline.

To circumvent this problem, we synthesized the compound analogous to **16** that bears the less acid-sensitive diethyl phosphonate group **19**. A procedure identical to that established for the dimethyl derivative but using triethyl phosphite as the phosphorus source provided the desired diethyl *N*-Cbz-1,2-dihydroisoquinoline-1-phosphonate **19** in 98% yield from isoquinoline. Taking into account the fact that compound **19** can be considered not only as an *N*-acyl-α-aminophosphonate, but also as an enamide, we decided to perform the reduction of the double bond using trifluoroacetic acid and triethylsilane as the reducing agent, following the conditions described by Jacobsen et al. [51] for cyclic enamides. In this case, the diethyl *N*-Cbz-1,2,3,4-tetrahydroisoquinoline-1-phosphonate **20** was readily obtained in quantitative yield by reaction with triethylsilane and trifluoroacetic acid. Subsequent cleavage of the protecting groups in **20** by treatment with hydrogen bromide in acetic acid, afforded the 1,2,3,4-tetrahydroisoquinoline-1-phosphonic acid **5** as hydrobromide in quantitative yield. This compound was isolated in 97% overall yield from isoquinoline (Scheme 4).

**Scheme 5:**
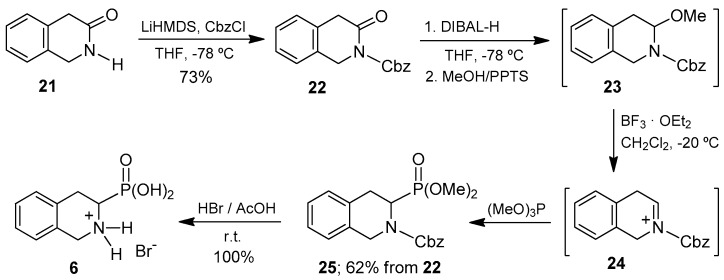
Synthesis of 1,2,3,4-tetrahydroisoquinoline-3-phosphonic acid, Tic^P^ **6**.

Finally, we focused on the preparation of 1,2,3,4-tetrahydroisoquinoline-3-phosphonic acid **6**, that is, the phosphonic analogue of Tic (Tic^P^). This compound exhibits a tetrahydroisoquinoline core, as does the aminophosphonic acid **5**. However, in the case of Tic^P^, the phosphonate group is at the 3-position. However, the isoquinoline selectively provides the phosphonate group at the 1-position of the ring. Therefore, we believe that the generation of a suitable iminium ion for the introduction of the phosphonate moiety at the desired 3-position, the lactam precursor was the only route to consider in this case. It should be noted that, following this strategy, the position of the carbonyl group in the starting lactam determines with complete regiocontrol the introduction of the phosphonate substituent, which is a distinctive advantage of this methodology. The adequate lactam **21** used in the synthesis of the target amino acid was easily prepared following reported procedures that involved reaction of 2-phenylacetyl chloride with aqueous ammonia to give 2-phenylacetamide [52] and subsequent condensation with formaldehyde [53]. Lactam **21** was readily transformed into 1,2,3,4-tetrahydroisoquinoline-3-phosphonic acid (Tic^P^, **6**) following the synthetic route strictly similar to that reported above for the preparation of the α-aminophosphonic acid **4** from lactam **7** (Scheme 1). The 1,2,3,4-tetrahydroisoquinoline-3-phosphonic acid **6** was thus obtained as hydrobromide in 52% global yield from the starting substrate **21**, with isolation and purification of only two synthetic intermediates **22**, **25** (Scheme 5).

The results obtained show the successful use of the lactam **21** to generate the key intermediate *N*-acyliminium ion, which by phosphonylation, afforded the desired diethyl *N*-Cbz-1,2,3,4-tetrahydroisoquinoline-3-phosphonate **25**, demonstrating thus the versatility of this methodology in the preparation of α-aminophosphonic acids structurally related to pipecolic acid.

## 3. Materials and Methods

### 3.1. General

All reagents were used as received from commercial suppliers (Sigma-Aldrich Chemie GmbH, Buchs, Switzerland) without further purification. Thin-layer chromatography (TLC) was performed on Macherey-Nagel Polygram^®^ SIL G/UV_254_ (Macherey-Nagel, Duren, Germany) precoated silica gel polyester plates. The products were visualized by exposure to UV light (254 nm), iodine vapour or ethanolic solution of phosphomolybdic acid. Column chromatography was performed using 60 M (0.04–0.063 mm) silica gel from Macherey-Nagel. Melting points were determined on a Gallenkamp apparatus (Weiss Gallenkamp, Loughborough, UK). IR spectra were registered on a Nicolet Avatar 360 FTIR spectrophotometer (Thermo Electron Corporation, Madison, WI, USA); ν_max_ is given for the main absorption bands. ^1^H-, ^13^C- and ^31^P-NMR spectra were recorded on Bruker AV-400 or AV-300 instruments (Bruker BioSpin GmbH, Rheinstetten, Germany) at room temperature, unless otherwise indicated, using the residual solvent signal as the internal standard; chemical shifts (δ) are expressed in ppm and coupling constants (*J*) in Hertz. High-resolution mass spectra were obtained on a Bruker Microtof-Q spectrometer. Compound **22** was prepared by reaction of 2-phenylacetamide [52] with formaldehyde [53]. ^1^H-, ^13^C- and ^31^P- NMR spectra of all final compounds are showed in the supplementary material (Figures S1–S34).

### 3.2. Synthesis of N-Benzyloxycarbonyl-3,4-dihydro-2-quinolinone ***8***

A 1 M solution of lithium bis(trimethylsilyl)amide in tetrahydrofuran (2.80 mL, 2.80 mmol) was slowly added to a solution of 3,4-dihydro-2(1*H*)-quinolinone **7** (400 mg, 2.72 mmol) in anhydrous tetrahydrofuran (10 mL) kept at −78 °C under argon. After 30 min, benzyl chloroformate (0.39 mL, 464 mg, 2.72 mmol) was added dropwise and stirring was continued for additional 3 h. The reaction mixture was then treated with saturated aqueous ammonium chloride (10 mL) and allowed to warm to room temperature. The two layers were separated and the aqueous phase was extracted with dichloromethane (2 × 20 mL). The combined organic extracts were dried, filtered, and concentrated. Purification by column chromatography (eluent:hexanes/ethyl acetate 4:1) afforded **8** as a colourless oil (694 mg, 2.47 mmol, 91% yield). IR (neat) ν_max_ 1773, 1699 cm^−1^. ^1^H-NMR (400 MHz, CDCl_3_): δ = 7.48–7.33 (m, 5H, Ar), 7.20–7.13 (m, 2H, Ar), 7.10–7.04 (m, 1H, Ar), 6.92–6.88 (m, 1H, Ar), 5.41 (s, 2H, C*H*_2_Ph), 2.98–2.92 (m, 2H, H-4), 2.72–2.67 (m, 2H, H-3) ppm. ^13^C-NMR (100 MHz, CDCl_3_): δ = 169.90 (CO), 153.46 (COO), 136.94 (Ar), 134.52 (Ar), 128.85 (Ar), 128.76 (Ar), 128.75 (Ar), 127.95 (Ar), 127.39 (Ar), 126.95 (Ar), 124.78 (Ar), 118.65 (Ar), 70.05 (*C*H_2_Ph), 33.05 (C-3), 25.55 (C-4) ppm. HRMS (ESI): calcd. for C_17_H_15_NNaO_3_ [M + Na]^+^ 304.0944; found 304.0947.

### 3.3. Synthesis of Dimethyl N-benzyloxycarbonyl-1,2,3,4-tetrahydroquinoline-2-phosphonate ***11***

A 1 M solution of diisobutylaluminium hydride in hexanes (2.40 mL, 2.40 mmol) was slowly added to a solution of **8** (448 mg, 1.59 mmol) in anhydrous tetrahydrofuran (8 mL) kept at −78 °C under argon. After stirring at this temperature for 2 h, the reaction was treated with saturated aqueous sodium acetate (5 mL) and warmed to room temperature. A 3:1 mixture of diethyl ether and saturated aqueous ammonium chloride (16 mL) was then added and the resulting mixture was stirred at room temperature until a suspension was formed. The solid was filtered off under reduced pressure and washed with diethyl ether (2 × 10 mL). The organic layer was separated and the aqueous phase was extracted with diethyl ether (2 × 20 mL). The combined organic extracts were washed with brine (20 mL), dried, filtered, and evaporated to provide the hemiaminal as an oil. It was dissolved in methanol (6 mL) and treated with pyridinium *p*-toluenesulfonate (40 mg, 0.16 mmol). After stirring at room temperature for 2 h, triethylamine (0.10 mL, 74 mg, 0.73 mmol) was added. The solvent was evaporated and the crude methoxyaminal **9** thus obtained was dissolved in anhydrous dichloromethane (7 mL) and kept under argon. Trimethyl phosphite (0.19 mL, 198 mg, 1.59 mmol) was added and the resulting solution was cooled to −20 °C. Boron trifluoride-diethyl ether (0.20 mL, 226 mg, 1.59 mmol) was added dropwise and the reaction mixture was slowly warmed to room temperature and stirred for 12 h. After quenching with water (2 mL), the two layers were separated and the aqueous phase was extracted with dichloromethane (2 × 10 mL). The combined organic extracts were dried, filtered, and concentrated. Purification by column chromatography (eluent:ethyl acetate/hexanes 4:1) afforded **11** as a colourless oil (372 mg, 0.99 mmol, 62% yield). IR (neat) ν_max_ 1702, 1279, 1029 cm^−1^. ^1^H-NMR (400 MHz, CDCl_3_): δ = 7.53–7.46 (m, 1H, Ar), 7.37–7.28 (m, 5H, Ar), 7.22–7.16 (m, 1H, Ar), 7.14–7.06 (m, 2H, Ar), 5.31 (d, *J =* 12.5 Hz, 1H, C*H*_2_Ph), 5.16 (d, *J =* 12.5 Hz, 1H, C*H*_2_Ph), 5.04 (ddd, *J =* 13.4, 8.7, 7.3 Hz, 1H, H-2), 3.63 (d, *J =* 10.6 Hz, 3H, OMe), 3.51 (d, *J =* 10.6 Hz, 3H, OMe), 2.85–2.75 (m, 1H, H-4), 2.67–2.57 (m, 1H, H-4′), 2.54–2.40 (m, 1H, H-3), 2.17–2.03 (m, 1H, H-3′) ppm. ^13^C-NMR (100 MHz, CDCl_3_): δ = 154.68 (CO), 136.84 (Ar), 136.08 (Ar), 132.78 (Ar), 128.54 (Ar), 128.16 (Ar), 127.88 (Ar), 127.56 (Ar), 126.36 (Ar), 125.97 (Ar), 125.22 (Ar), 68.11 (*C*H_2_Ph), 53.07 (d, *J =* 6.2 Hz, OMe), 52.82 (d, *J =* 7.2 Hz, OMe), 49.33 (d, *J =* 158.2 Hz, C-2), 25.75 (C-3), 25.66 (C-4) ppm. ^31^P-NMR (162 MHz, CDCl_3_): δ = 26.39 ppm. HRMS (ESI): calcd. for C_19_H_22_NNaO_5_P [M + Na]^+^ 398.1128; found 398.1145.

### 3.4. Synthesis of Dimethyl N-Benzyloxycarbonyl-1,2-dihydroquinoline-2-phosphonate ***13***

Benzyl chloroformate (0.61 mL, 726 mg, 4.26 mmol) was added dropwise to a solution of quinoline (0.46 mL, 500 mg, 3.87 mmol) in anhydrous acetonitrile (6 mL) kept at 0 °C under argon. After stirring at this temperature for 10 min, trimethyl phosphite (0.50 mL, 528 mg, 4.26 mmol) was slowly added followed by sodium iodide (853 mg, 5.69 mmol). The mixture was heated at 50 °C for 10 min. The solvent was evaporated and the crude product was partitioned between dichloromethane (15 mL) and saturated aqueous sodium bicarbonate (15 mL). The organic phase was separated and the aqueous layer was further extracted with dichloromethane (3 × 15 mL). The combined organic extracts were washed with brine (10 mL), dried, filtered, and concentrated. Purification by column chromatography (eluent:ethyl acetate/hexanes 4:1) afforded **13** as a colourless oil (1.07 g, 2.87 mmol, 74% yield). IR (neat) ν_max_ 1706, 1653, 1603, 1263, 1125, 1026 cm^−1^. ^1^H-NMR (400 MHz, DMSO-*d*_6_, 80 °C): δ = 7.58–7.52 (m, 1H, Ar), 7.44–7.30 (m, 5H, Ar), 7.25–7.07 (m, 3H, Ar), 6.71–6.65 (m, 1H, H-4), 6.07 (ddd, *J =* 9.5, 6.3, 4.5 Hz, 1H, H-3), 5.61 (ddd, *J =* 21.4, 6.3, 1.2 Hz, 1H, H-2), 5.27 (s, 2H, C*H*_2_Ph), 3.47 (d, *J =* 10.6 Hz, 6H, OMe) ppm. ^13^C-NMR (100 MHz, DMSO-*d*_6_, 80 °C): δ = 152.90 (d, *J =* 4.7 Hz, CO), 135.68 (Ar), 134.66 (Ar), 128.05 (Ar), 127.70 (Ar), 127.40 (Ar), 127.35 (Ar), 127.01 (d, *J* = 9.7 Hz, C-4), 126.77 (d, *J =* 4.0 Hz, Ar), 126.06 (d, *J =* 1.5 Hz, Ar), 124.35 (Ar), 123.66 (Ar), 122.55 (d, *J =* 3.0 Hz, C-3), 67.44 (*C*H_2_Ph), 52.70 (d, *J =* 7.0 Hz, OMe), 52.39 (d, *J =* 6.6 Hz, OMe), 50.84 (d, *J =* 151.5 Hz, C-2) ppm. ^31^P-NMR (162 MHz, DMSO-*d*_6_, 80 °C): δ = 20.41 ppm. HRMS (ESI): calcd. for C_19_H_20_NNaO_5_P [M + Na]^+^ 396.0971; found 396.0991.

### 3.5. Synthesis of Dimethyl 1,2,3,4-Tetrahydroquinoline-2-phosphonate ***14***

A mixture of **13** (200 mg, 0.54 mmol) and 10% Pd/C (20 mg) in ethyl acetate (10 mL) was stirred at room temperature under an atmospheric pressure of hydrogen gas for 12 h. Filtration of the catalyst and evaporation of the solvent provided pure **14** as a colourless oil (130 mg, 0.54 mmol, 100% yield). IR (neat) ν_max_ 3316, 1231, 1057, 1029 cm^−1^. ^1^H-NMR (400 MHz, CDCl_3_): δ = 7.02–6.93 (m, 2H, Ar), 6.70–6.63 (m, 1H, Ar), 6.58–6.53 (m, 1H, Ar), 4.16 (br s, 1H, NH), 3.82 (d, *J =* 10.3 Hz, 3H, OMe), 3.81 (d, *J =* 10.5 Hz, 3H, OMe), 3.71 (ddd, *J =* 10.1, 6.7, 3.4 Hz, 1H, H-2), 2.88–2.75 (m, 2H, H-4), 2.29–2.19 (m, 1H, H-3), 2.12–1.98 (m, 1H, H-3′) ppm. ^13^C-NMR (100 MHz, CDCl_3_): δ = 143.08 (d, *J =* 10.9 Hz, Ar), 129.40 (Ar), 127.11 (Ar), 121.05 (Ar), 118.26 (Ar), 114.97 (Ar), 53.83 (d, *J =* 6.7 Hz, OMe), 53.08 (d, *J =* 7.3 Hz, OMe), 49.05 (d, *J =* 161.5 Hz, C-2), 26.09 (d, *J =* 13.7 Hz, C-4), 22.39 (d, *J =* 5.2 Hz, C-3) ppm. ^31^P-NMR (162 MHz, CDCl_3_): δ = 27.39 ppm. HRMS (ESI): calcd. for C_11_H_16_NNaO_3_P [M + Na]^+^ 264.0760; found 264.0752.

### 3.6. Synthesis of 1,2,3,4-Tetrahydroquinoline-2-phosphonic Acid Hydrobromide ***4***

From **11**: A 33% solution of hydrogen bromide in acetic acid (2 mL) was added to compound **11** (110 mg, 0.29 mmol) and the reaction mixture was stirred at room temperature for 3 h. The solvent was evaporated and the residue was taken up in water and lyophilised to afford **4** as a white solid (86 mg, 0.29 mmol, 100% yield). M.p. 94–96 °C (dec.). IR (nujol) ν_max_ 3381, 1171, 1077, 1057 cm^−1^. ^1^H-NMR (400 MHz, CD_3_OD): δ = 7.40–7.28 (m, 4H, Ar), 3.78 (ddd, *J* = 13.1, 12.1, 2.5 Hz, 1H, H-2), 3.08–3.01 (m, 2H, H-4), 2.52–2.43 (m, 1H, H-3), 2.19–2.06 (m, 1H, H-3′) ppm. ^13^C-NMR (100 MHz, CD_3_OD): δ = 132.21 (d, *J* = 0.7 Hz, Ar), 131.87 (Ar), 131.78 (Ar), 130.25 (Ar), 128.64 (Ar), 124.49 (Ar), 52.52 (d, *J* = 153.2 Hz, C-2), 25.94 (d, *J* = 12.4 Hz, C-4), 22.37 (d, *J* = 2.2 Hz, C-3) ppm. ^31^P-NMR (162 MHz, CD_3_OD): δ = 14.61 ppm. HRMS (ESI): calcd. for C_9_H_13_NO_3_P [M − Br]^+^ 214.0628; found 214.0632.

From **14**: A 33% solution of hydrogen bromide in acetic acid (2 mL) was added to compound **14** (130 mg, 0.54 mmol) and the reaction mixture was stirred at room temperature for 3 h. The solvent was evaporated and the residue was taken up in water and lyophilised to afford **4** as a white solid (158 mg, 0.54 mmol, 100% yield). Spectroscopic data were identical to those described above.

### 3.7. Synthesis of Dimethyl N-Benzyloxycarbonyl-1,2-dihydroisoquinoline-1-phosphonate ***16***

Benzyl chloroformate (0.61 mL, 726 mg, 4.26 mmol) was added dropwise to a solution of isoquinoline (500 mg, 3.87 mmol) and trimethyl phosphite (0.50 mL, 528 mg, 4.26 mmol) in anhydrous acetonitrile (6 mL) kept at 0 °C under argon. Sodium iodide (853 mg, 5.69 mmol) was slowly added and the mixture was heated at 50 °C for 10 min. The solvent was evaporated and the crude product was partitioned between dichloromethane (15 mL) and saturated aqueous sodium bicarbonate (15 mL). The organic phase was separated and the aqueous layer was further extracted with dichloromethane (3 × 15 mL). The combined organic extracts were washed with brine (10 mL), dried, filtered, and concentrated. Purification by column chromatography (eluent: ethyl acetate/hexanes 4:1) afforded **16** as a colourless oil (1.33 g, 3.56 mmol, 92% yield). IR (neat) ν_max_ 1715, 1342, 1295, 1238, 1121, 1028 cm^−1^. ^1^H-NMR (300 MHz, DMSO-*d*_6_, 50 °C): δ = 7.51–7.11 (m, 9H, Ar), 6.91 (d, *J* = 7.7 Hz, 1H, H-3), 6.08–5.94 (m, 1H, H-4), 5.83 (d, *J* = 15.8 Hz, 1H, H-1), 5.27 (s, 2H, C*H*_2_Ph), 3.57–3.38 (m, 6H, OMe) ppm. ^13^C-NMR (100 MHz, DMSO-*d*_6_): δ = (duplicate signals are observed for some carbons; asterisks indicate those corresponding to the minor rotamer) 152.37* (CO), 151.88 (CO), 136.01 (Ar), 135.91* (Ar), 131.03* (d, *J* = 3.6 Hz, Ar), 130.84 (d, *J* = 3.6 Hz, Ar), 128.75* (d *J* = 3.0 Hz, Ar), 128.68 (d, *J* = 3.1 Hz, Ar), 128.54 (Ar), 128.47* (Ar), 128.26 (Ar), 128.11* (Ar), 127.92 (Ar), 127.55 (d, *J* = 5.2 Hz, Ar), 127.30 (d, *J* = 2.3 Hz, Ar), 127.18 (d, *J* = 2.0 Hz, Ar), 125.43 (Ar), 125.35 (Ar), 124.86 (d, *J* = 2.9 Hz, C-3), 124.79* (C-3), 109.86 (C-4), 67.86* (*C*H_2_Ph), 67.77 (*C*H_2_Ph), 53.40* (d, *J* = 148.4 Hz, C-1), 53.16* (d, *J* = 6.1 Hz, OMe), 53.12 (d, *J* = 7.0 Hz, OMe), 52.64 (d, *J* = 149.6 Hz, C-1) ppm. ^31^P-NMR (122 MHz, DMSO-*d*_6_, 50 °C): δ = 21.34 ppm. HRMS (ESI): calcd. for C_19_H_20_NNaO_5_P [M + Na]^+^ 396.0971; found 396.0982.

### 3.8. Synthesis of Diethyl N-Benzyloxycarbonyl-1,2-dihydroisoquinoline-1-phosphonate ***19***

Benzyl chloroformate (0.61 mL, 726 mg, 4.26 mmol) was added dropwise to a solution of isoquinoline (500 mg, 3.87 mmol) and triethyl phosphite (0.73 mL, 708 mg, 4.26 mmol) in anhydrous acetonitrile (6 mL) kept at 0 °C under argon. Sodium iodide (853 mg, 5.69 mmol) was slowly added and the mixture was heated at 50 °C for 10 min. The solvent was evaporated and the crude product was partitioned between dichloromethane (15 mL) and saturated aqueous sodium bicarbonate (15 mL). The organic phase was separated and the aqueous layer was further extracted with dichloromethane (3 × 15 mL). The combined organic extracts were washed with brine (10 mL), dried, filtered, and concentrated. Purification by column chromatography (eluent:ethyl acetate/hexanes 3:2) afforded **19** as a colourless oil (1.52 g, 3.79 mmol, 98% yield). IR (neat) ν_max_ 1715, 1294, 1253, 1120, 1023 cm^−1^. ^1^H-NMR (300 MHz, DMSO-*d*_6_, 70 °C): δ = 7.50–7.10 (m, 9H, Ar), 6.91 (d, *J* = 7.8 Hz, 1H, H-3), 5.98 (d, *J* = 7.8 Hz, 1H, H-4), 5.77 (d, *J* = 15.9 Hz, 1H, H-1), 5.26 (s, 2H, C*H*_2_Ph), 3.95–3.69 (m, 4H, OCH_2_), 1.08 (t, *J* = 7.0 Hz, 3H, Me), 1.07 (t, *J* = 7.0 Hz, 3H, Me) ppm. ^13^C-NMR (100 MHz, DMSO-*d*_6_): δ = (duplicate signals are observed for some carbons; asterisks indicate those corresponding to the minor rotamer) 152.48* (CO), 151.91 (CO), 136.05 (Ar), 135.88* (Ar), 131.15* (d, *J* = 3.6 Hz, Ar), 130.98 (d, *J* = 3.7 Hz, Ar), 128.69* (d, *J* = 3.3 Hz, Ar), 128.61 (d, *J* = 3.3 Hz, Ar), 128.56 (Ar), 128.47* (Ar), 128.28 (Ar), 128.25* (Ar), 128.13* (Ar), 127.97 (Ar), 127.53 (d, *J* = 5.2 Hz, Ar), 127.22 (d, *J* = 2.6 Hz, Ar), 127.11* (d, *J* = 2.7 Hz, Ar), 125.59 (d, *J* = 1.8 Hz, Ar), 125.56* (d, *J* = 2.4 Hz, Ar), 125.42 (Ar), 124.84* (C-3), 124.81 (C-3), 109.95 (C-4), 67.84* (*C*H_2_Ph), 67.71 (*C*H_2_Ph), 62.54 (d, *J* = 7.1 Hz, OCH_2_), 62.42* (d, *J* = 6.6 Hz, OCH_2_), 62.38* (d, *J* = 7.1 Hz, OCH_2_), 53.97* (d, *J* = 148.9 Hz, C-1), 53.15 (d, *J* = 150.2 Hz, C-1), 16.17 (d, *J* = 5.3 Hz, Me), 16.15* (d, *J* = 5.7 Hz, Me) ppm. ^31^P-NMR (122 MHz, DMSO-*d*_6_, 70 °C): δ = 18.77 ppm. HRMS (ESI): calcd. for C_21_H_25_NO_5_P [M + H]^+^ 402.1465; found 402.1466.

### 3.9. Synthesis of Diethyl N-Benzyloxycarbonyl-1,2,3,4-tetrahydroisoquinoline-1-phosphonate ***20***

Triethylsilane (1.0 mL, 730 mg, 6.28 mmol) and trifluoroacetic acid (0.48 mL, 716 mg, 6.28 mmol) were added to a solution of **19** (300 mg, 0.75 mmol) in anhydrous dichloromethane (15 mL) kept at 0 °C under argon. The solution was allowed to warm to room temperature and stirred for 18 h. Evaporation of the solvent followed by column chromatography (eluent:hexanes/ethyl acetate 1:1) afforded **20** as a colourless oil (298 mg, 0.74 mmol, 99% yield). IR (neat) ν_max_ 1701, 1294, 1249, 1230, 1051, 1022 cm^−1^. ^1^H-NMR (300 MHz, DMSO-*d*_6_, 70 °C): δ = 7.44–7.16 (m, 9H, Ar), 5.53 (d, *J* = 20.4 Hz, 1H, H-1), 5.17 (s, 2H, C*H*_2_Ph), 4.14–3.77 (m, 5H, H-3, OCH_2_), 3.72–3.52 (m, 1H, H-3′), 2.98–2.78 (m, 2H, H-4), 1.17 (t, *J* = 7.0 Hz, 3H, Me), 1.08 (t, *J* = 6.9 Hz, 3H, Me) ppm. ^13^C-NMR (100 MHz, DMSO-*d*_6_): δ = (duplicate signals are observed for some carbons; asterisks indicate those corresponding to the minor rotamer) 154.60 (d, *J* = 3.9 Hz, CO), 154.18* (d, *J* = 2.4 Hz, CO), 136.66 (Ar), 136.46* (Ar), 134.73 (d, *J* = 5.6 Hz, Ar), 134.63* (d, *J* = 5.6 Hz, Ar), 129.22 (Ar), 129.13* (d, *J* = 2.2 Hz, Ar), 128.95 (d, *J* = 2.3 Hz, Ar), 128.85 (Ar), 128.38 (Ar), 128.33* (Ar), 127.94* (Ar), 127.91 (Ar), 127.86* (Ar), 127.67 (d, *J* = 4.0 Hz, Ar), 127.58 (Ar), 127.38 (Ar), 125.88 (d, *J* = 2.8 Hz, Ar), 125.83* (d, *J* = 2.8 Hz, Ar), 66.92* (*C*H_2_Ph), 66.82 (*C*H_2_Ph), 62.58 (d, *J* = 7.2 Hz, OCH_2_), 62.19 (d, *J* = 7.1 Hz, OCH_2_), 52.76* (d, *J* = 149.9 Hz, C-1), 52.34 (d, *J* = 152.0 Hz, C-1), 39.05* (C-3), 38.84 (C-3), 27.43 (C-4), 27.15* (C-4), 16.08* (d, *J* = 5.6 Hz, Me), 16.05 (d, *J* = 5.3 Hz, Me). ^31^P-NMR (122 MHz, DMSO-*d*_6_, 70 °C): δ = 20.75. HRMS (ESI): calcd. for C_21_H_27_NO_5_P [M + H]^+^ 404.1621; found 404.1611.

### 3.10. Synthesis of 1,2,3,4-Tetrahydroisoquinoline-1-phosphonic Acid Hydrobromide ***5***

A 33% solution of hydrogen bromide in acetic acid (2 mL) was added to **20** (160 mg, 0.40 mmol) and the reaction mixture was stirred at room temperature for 3 h. The solvent was evaporated and the residue was taken up in water and lyophilised to afford **5** as a white solid (117 mg, 0.40 mmol, 100% yield). M.p. 85–87 °C (dec.). IR (nujol) ν_max_ 3421, 1212, 1118, 1019 cm^−1^. ^1^H-NMR (400 MHz, D_2_O): δ = 7.45–7.41 (m, 1H, Ar), 7.37–7.27 (m, 3H, Ar), 4.70 (d, *J* = 17.6 Hz, 1H, H-1), 3.78 (ddd, *J* = 12.7, 9.2, 6.5 Hz, 1H, H-3), 3.58–3.51 (m, 1H, H-3′), 3.18–3.12 (m, 2H, H-4) ppm. ^13^C-NMR (100 MHz, CD_3_OD): δ = 132.88 (d, *J* = 5.4 Hz, Ar), 130.40 (d, *J* = 2.2 Hz, Ar), 129.47 (d, *J* = 2.7 Hz, Ar), 128.94 (d, *J* = 3.6 Hz, Ar), 127.92 (d, *J* = 2.6 Hz, Ar), 127.40 (d, *J* = 5.1 Hz, Ar), 54.00 (d, *J* = 147.3 Hz, C-1), 41.15 (d, *J* = 2.1 Hz, C-3), 25.88 (C-4) ppm. ^31^P-NMR (162 MHz, D_2_O): δ = 10.02 ppm. HRMS (ESI): calcd. for C_9_H_13_NO_3_P [M − Br]^+^ 214.0628; found 214.0633.

### 3.11. Synthesis of N-Benzyloxycarbonyl-1,4-dihydro-3-isoquinolinone ***22***

A 1 M solution of lithium bis(trimethylsilyl)amide in tetrahydrofuran (0.83 mL, 0.83 mmol) was slowly added to a solution of 1,4-dihydro-3(2*H*)-isoquinolinone **21** (122 mg, 0.83 mmol) in anhydrous tetrahydrofuran (5 mL) kept at −78 °C under argon. After 30 min, benzyl chloroformate (0.12 mL, 142 mg, 0.83 mmol) was added dropwise and stirring was continued for additional 3 h. The reaction was then treated with saturated aqueous ammonium chloride (10 mL) and allowed to warm to room temperature. The two layers were separated and the aqueous phase was extracted with dichloromethane (2 × 10 mL). The combined organic extracts were dried, filtered, and concentrated. Purification by column chromatography (eluent:hexanes/ethyl acetate 7:3) afforded **22** as a white solid (172 mg, 0.61 mmol, 73% yield). M.p. 63–65 °C. IR (nujol) ν_max_ 1692 cm^−1^. ^1^H-NMR (400 MHz, CDCl_3_): δ = 7.49–7.44 (m, 2H, Ar), 7.40–7.25 (m, 6H, Ar), 7.23–7.19 (m, 1H, Ar), 5.34 (s, 2H, C*H*_2_Ph), 4.92 (s, 2H, H-1), 3.73 (s, 2H, H-4) ppm. ^13^C-NMR (100 MHz, CDCl_3_): δ = 169.43 (CO), 153.62 (COO), 135.38 (Ar), 132.25 (Ar), 132.13 (Ar), 128.74 (Ar), 128.50 (Ar), 128.46 (Ar), 128.22 (Ar), 127.35 (Ar), 126.99 (Ar), 125.81 (Ar), 68.97 (*C*H_2_Ph), 48.75 (C-1), 41.53 (C-4) ppm. HRMS (ESI): calcd. for C_17_H_15_NNaO_3_ [M + Na]^+^ 304.0944; found 304.0943.

### 3.12. Synthesis of Dimethyl N-Benzyloxycarbonyl-1,2,3,4-tetrahydroisoquinoline-3-phosphonate ***25***

A 1 M solution of diisobutylaluminium hydride in hexanes (0.78 mL, 0.78 mmol) was slowly added to a solution of **22** (146 mg, 0.52 mmol) in anhydrous tetrahydrofuran (5 mL) kept at −78 °C under argon. After stirring at this temperature for 2 h, the reaction was treated with saturated aqueous sodium acetate (10 mL) and allowed to warm to room temperature. A 3:1 mixture of diethyl ether and saturated aqueous ammonium chloride (16 mL) was then added and the resulting mixture was stirred at room temperature until a suspension was formed. The solid was filtered off under reduced pressure and washed with diethyl ether (2 × 10 mL). The organic layer was separated and the aqueous phase was extracted with diethyl ether (2 × 10 mL). The combined organic extracts were washed with brine (10 mL), dried, filtered, and evaporated to provide the hemiaminal as an oil. It was dissolved in methanol (5 mL) and treated with pyridinium *p*-toluenesulfonate (13 mg, 0.05 mmol). After stirring at room temperature for 2 h, triethylamine (31 µL, 22 mg, 0.22 mmol) was added. The solvent was evaporated and the crude methoxyaminal obtained **23** was dissolved in anhydrous dichloromethane (5 mL) and kept under argon. Trimethyl phosphite (61 µL, 65 mg, 0.52 mmol) was added and the resulting solution was cooled to −20 °C. Boron trifluoride-diethyl ether (65 µL, 74 mg, 0.52 mmol) was added and the reaction mixture was slowly warmed to room temperature and stirred for 12 h. After quenching with water (10 mL), the two layers were separated and the aqueous phase was extracted with dichloromethane (2 × 10 mL). The combined organic extracts were dried, filtered, and concentrated. Purification by column chromatography (eluent:ethyl acetate/hexanes 9:1) afforded **25** as a colourless oil (138 mg, 0.37 mmol, 71% yield). IR (neat) ν_max_ 1703, 1410, 1246, 1055, 1031 cm^−1^. ^1^H-NMR (400 MHz, CDCl_3_): δ = (duplicate signals are observed for some protons; asterisks indicate those corresponding to the minor rotamer) 7.42–7.30 (m, 5H, Ar), 7.23–7.04 (m, 4H, Ar), 5.31–5.12 (m, 2H, C*H*_2_Ph), 5.11–5.03 (m, 1H, H-3), 5.00–4.86 (m, 1H, H-1) overlapped with 4.97*–4.89* (m, 1H, H-3), 4.52 (d, *J* = 16.6 Hz, 1H, H-1′), 4.45* (d, *J* = 16.6 Hz, 1H, H-1′), 3.65 (d, *J* = 10.7 Hz, 3H, OMe), 3.51* (d, *J* = 10.7 Hz, 3H, OMe), 3.35 (d, *J* = 10.7 Hz, 3H, OMe), 3.33–3.16 (m, 2H, H-4) overlapped with 3.24* (d, *J* = 10.7 Hz, 3H, OMe) ppm. ^13^C-NMR (100 MHz, CDCl_3_): δ = (duplicate signals are observed for some carbons; asterisks indicate those corresponding to the minor rotamer) 155.72 (CO), 155.05* (CO), 136.37 (Ar), 136.18* (Ar), 132.61* (Ar), 132.46 (Ar), 131.41 (Ar), 131.00* (Ar), 128.64 (Ar), 128.53 (Ar), 128.45* (Ar), 128.24 (Ar), 128.17* (Ar), 127.95 (Ar), 126.70 (Ar), 126.64* (Ar), 126.60 (Ar), 126.09* (Ar), 125.86 (Ar), 67.79 (*C*H_2_Ph), 52.74 (d, *J* = 6.4 Hz, OMe), 52.58 (d, *J* = 6.6 Hz, OMe), 52.34* (d, *J* = 6.9 Hz, OMe), 47.07* (d, *J* = 154.2 Hz, C-3), 46.29 (d, *J* = 154.1 Hz, C-3), 44.37 (C-1), 28.56* (C-4), 28.32 (C-4) ppm. ^31^P-NMR (162 MHz, CDCl_3_): δ = (a duplicate signal is observed; an asterisk indicates that corresponding to the minor rotamer) 27.67, 27.07* ppm. HRMS (ESI): calcd. for C_19_H_22_NNaO_5_P [M + Na]^+^ 398.1128; found 398.1144.

### 3.13. Synthesis of 1,2,3,4-Tetrahydroisoquinoline-3-phosphonic Acid Hydrobromide ***6***

A 33% solution of hydrogen bromide in acetic acid (2 mL) was added to **25** (100 mg, 0.27 mmol) and the reaction mixture was stirred at room temperature for 3 h. The solvent was evaporated and the residue was taken up in water and lyophilised to afford **6** as a white solid (79 mg, 0.27 mmol, 100% yield). M.p. 102–103 °C (dec.). IR (nujol) ν_max_ 3404, 1232, 1010 cm^−1^. ^1^H-NMR (400 MHz, CD_3_OD): δ = 7.35–7.22 (m, 4H, Ar), 4.52 (d, *J* = 15.7 Hz, 1H, H-1), 4.42 (dd, *J* = 15.7, 3.1 Hz, 1H, H-1′), 3.94–3.83 (m, 1H, H-3), 3.34–3.25 (m, 2H, H-4) ppm. ^13^C-NMR (100 MHz, CD_3_OD): δ = 131.71 (d, *J* = 12.2 Hz, Ar), 129.97 (Ar), 129.27 (Ar), 128.69 (Ar), 128.32 (Ar), 127.77 (Ar), 51.34 (d, *J* = 153.1 Hz, C-3), 47.01 (d, *J* = 7.7 Hz, C-1), 27.44 (C-4) ppm. ^31^P-NMR (162 MHz, CD_3_OD): δ = 14.06 ppm. HRMS (ESI): calcd. for C_9_H_13_NO_3_P [M − Br]^+^ 214.0628; found 214.0631.

## 4. Conclusions

The synthesis of α-aminophosphonic acids characterized by a tetrahydroquinoline or tetrahydroisoquinoline heterocycles is described. Specifically, 1,2,3,4-tetrahydroquinoline-2-phosphonic acid **4**, 1,2,3,4-tetrahydroisoquinoline-1-phosphonic acid **5**, and 1,2,3,4-tetrahydro-isoquinoline-3-phosphonic acid **6** have been prepared in high overall yields using efficient methods that make use of easily available substrates. The compounds obtained can be viewed as higher homologues of phosphopipecolic acid that bear a benzene ring fused at different positions of the six-membered piperidine cycle. In particular, the latter is the phosphonic surrogate of Tic, an amino acid of extraordinary value in the design of pharmacologically useful peptides. The synthetic routes developed rely on the addition of trialkyl phosphites to iminium ions generated from quinoline/isoquinoline or from the appropriate δ-lactam bearing a fused benzene ring. Thus, lactams have proven to be versatile starting materials for the efficient and selective synthesis of α-aminophosphonic acids containing an azacyclic skeleton. The presence of the carbonyl group in the starting lactam determines, with full regiocontrol, the position at which the phosphonate substituent is incorporated. Formation of the intermediate *N*-acyliminium ion with the adequate regiochemistry has allowed the synthesis of the phosphonic counterpart of Tic (Tic^P^). Importantly, this α-aminophosphonic acid is not accessible through other methodologies traditionally used to generate iminium ions.

## Supplementary Materials

^1^H-, ^13^C-, and ^31^P- (when applicable) NMR spectra of the final compounds and all key intermediates are available online at http://www.mdpi.com/1420-3049/21/9/1140/s1.
