# Supplementary material for: Practical and Efficient Synthesis of α-Aminophosphonic Acids Containing 1,2,3,4-Tetrahydroquinoline or 1,2,3,4-Tetrahydroisoquinoline Heterocycles
Source: Molecules. 2016 Aug 31;21(9):1140. doi: 10.3390/molecules21091140 (PMC6273407; doi:10.3390/molecules21091140)
Supplement: Supplementary file 1 [file molecules-21-01140-s001.pdf]

Supplementary Materials: Practical and Efficient Synthesis of α-Aminophosphonic Acids Containing 1,2,3,4-Tetrahydroquinoline or 1,2,3,4-Tetrahydroisoquinoline Heterocycles

Mario Ordóñez, Alicia Arizpe, Fracisco J. Sayago, Ana I. Jiménez and Carlos Cativiela

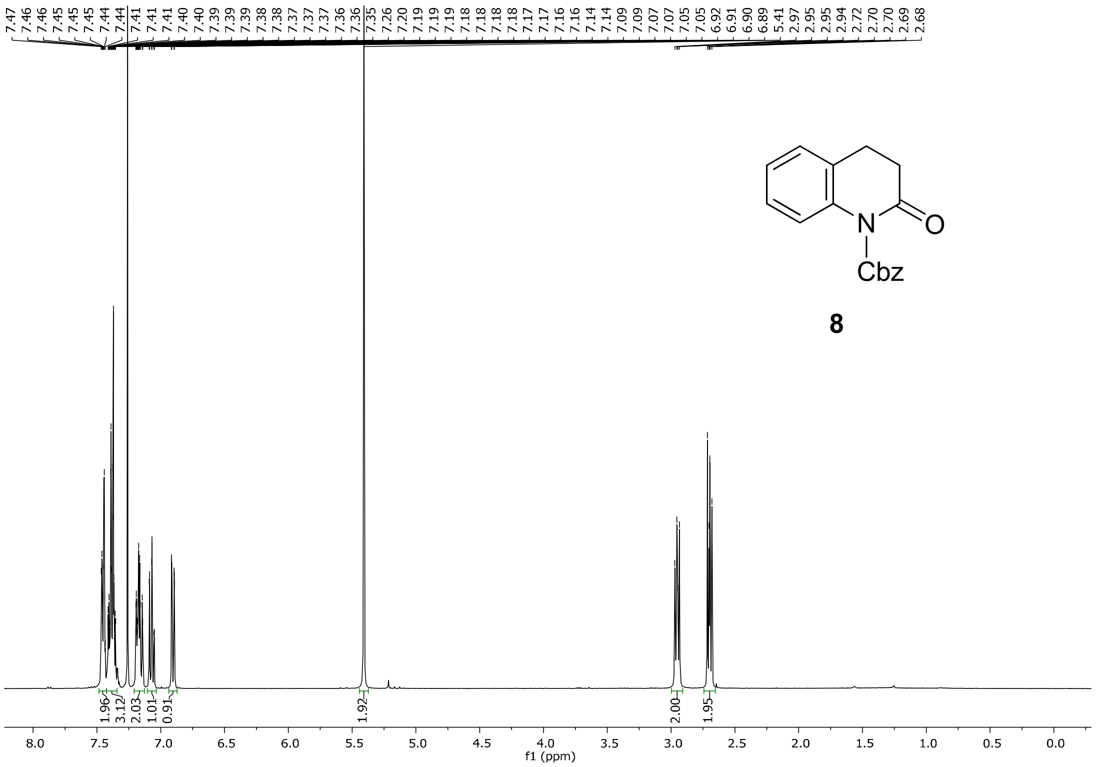

Figure S1. ^{1}H-NMR of **8** (400 MHz, $CDCl_3$).

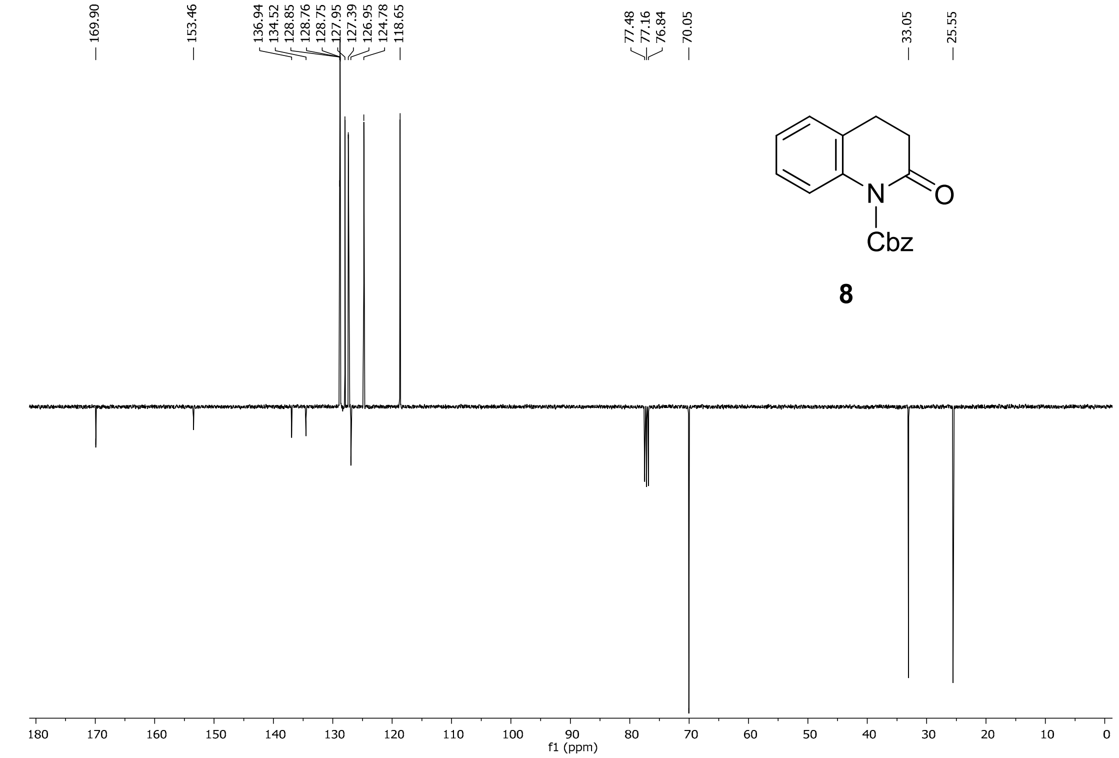

Figure S2. ^{13}C-NMR of **8** (100 MHz, $CDCl_3$).

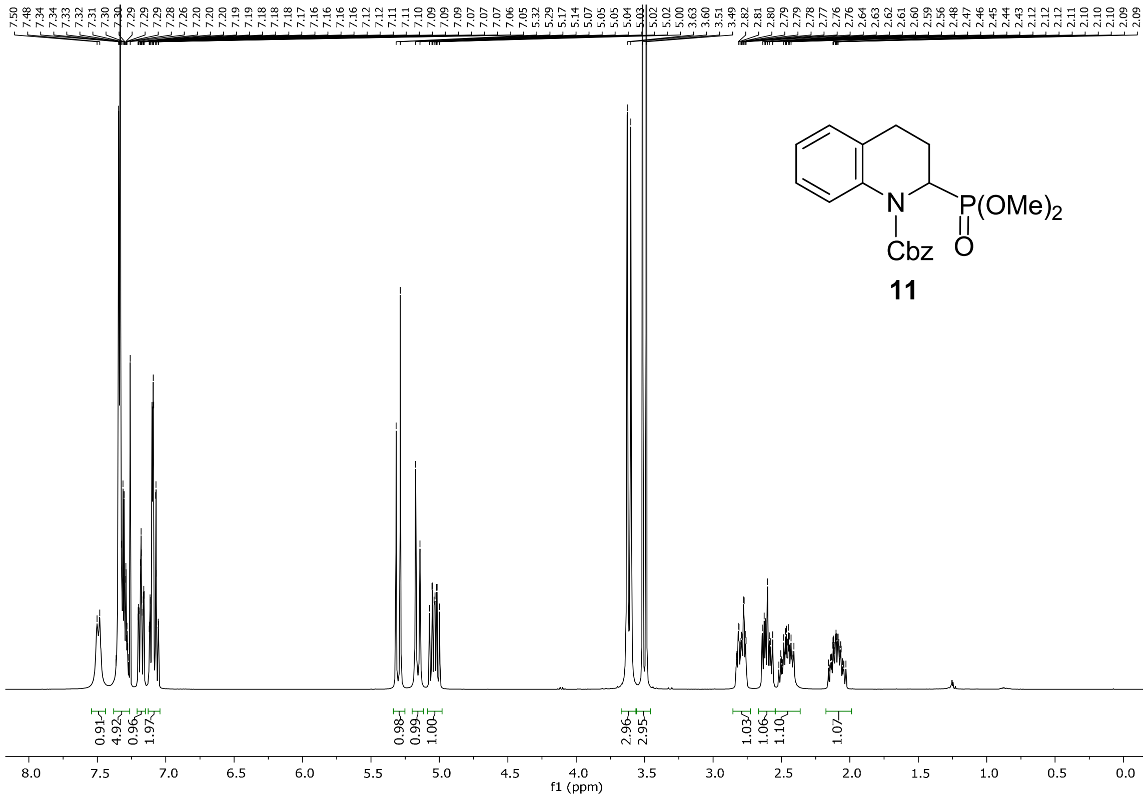

Figure S3. ^{1}H-NMR of **11** (400 MHz, $CDCl_3$).

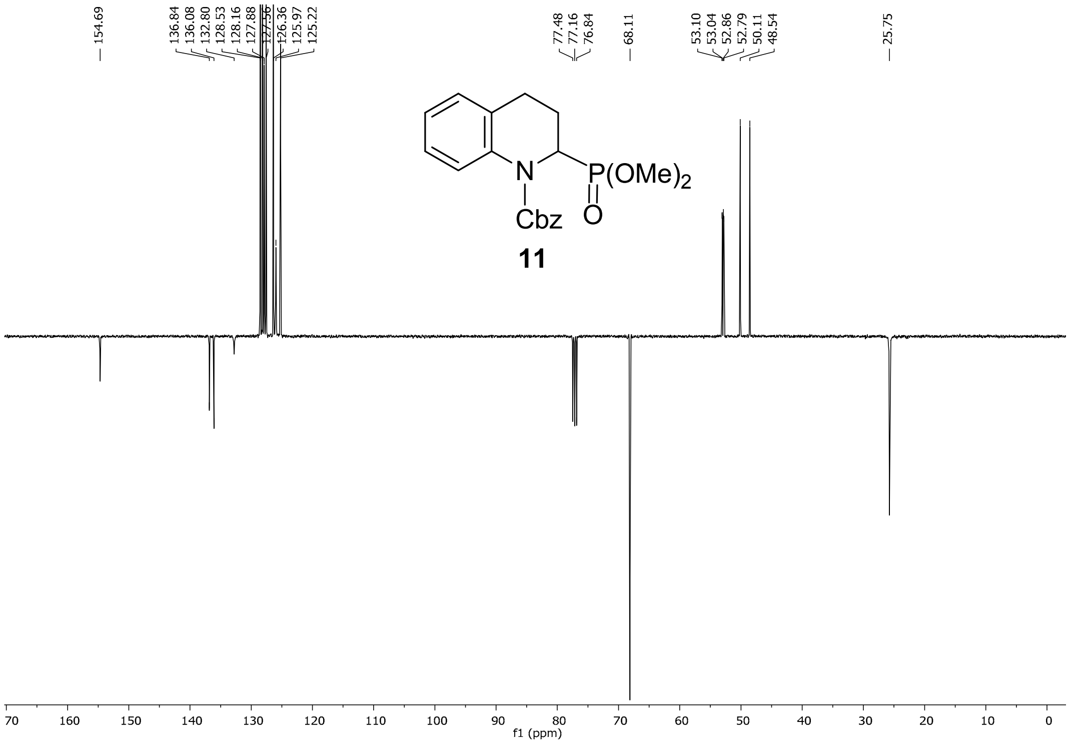

Figure S4. ^{13}C-NMR of **11** (100 MHz, $CDCl_3$).

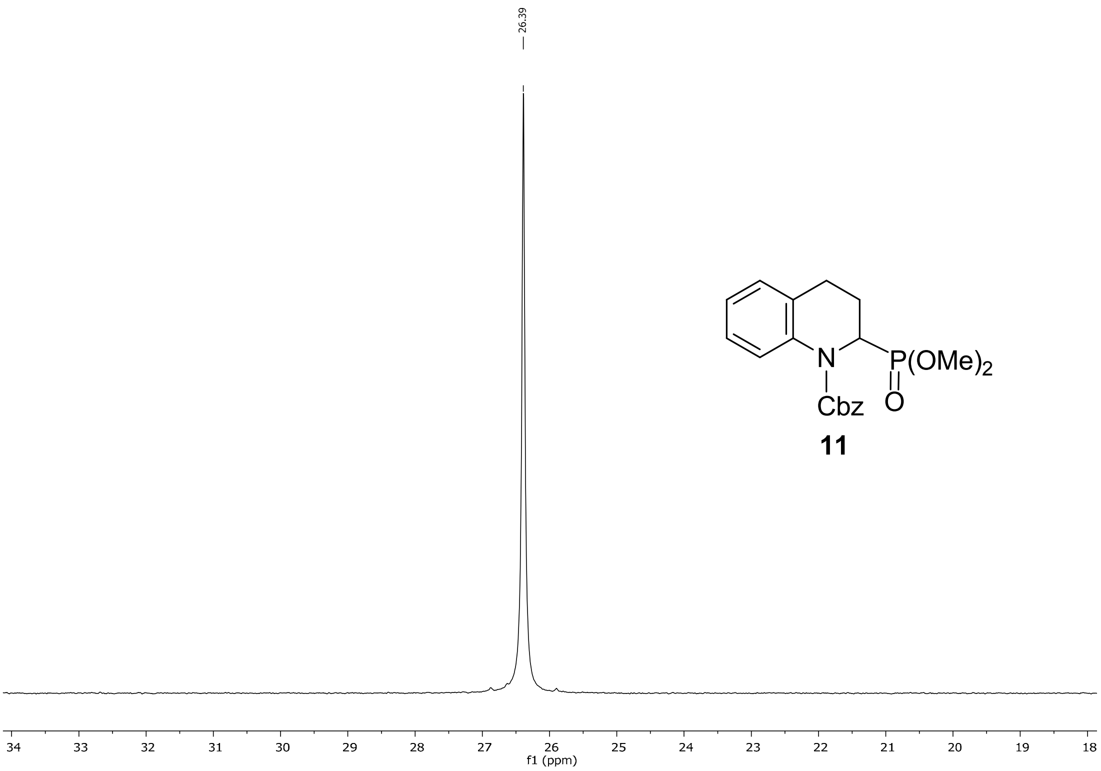

Figure S5. ^{31}P-NMR of **11** (162 MHz, $CDCl_3$).

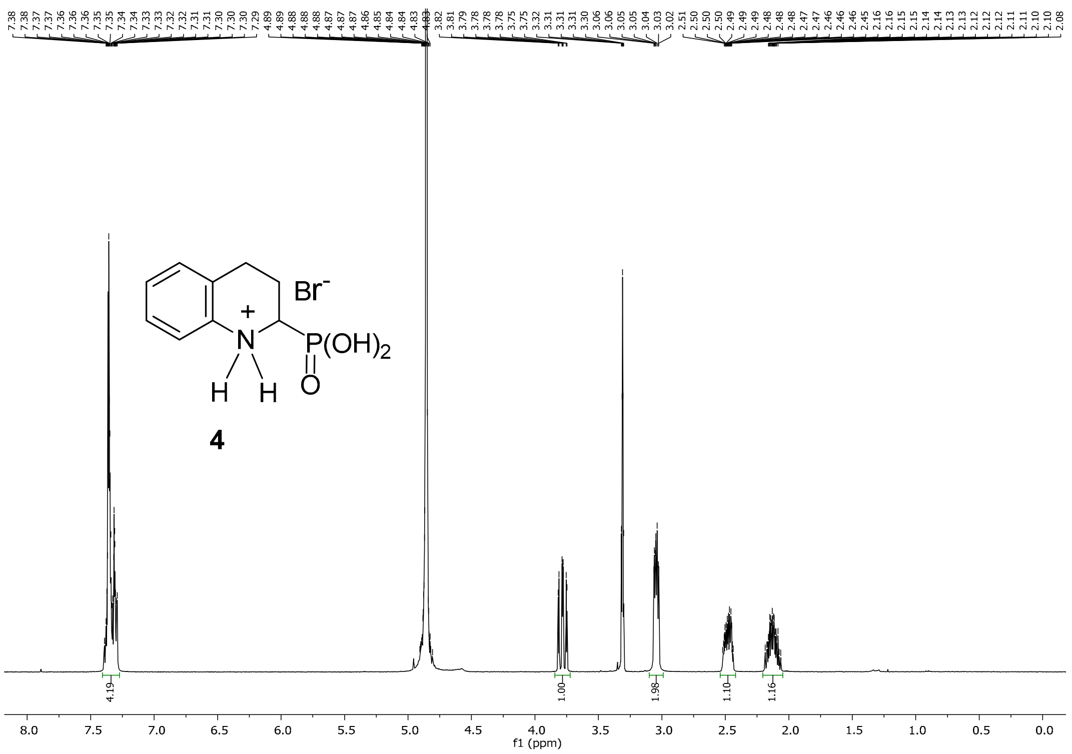

Figure S6. ^{1}H-NMR of **4** (400 MHz, CD_3OD).

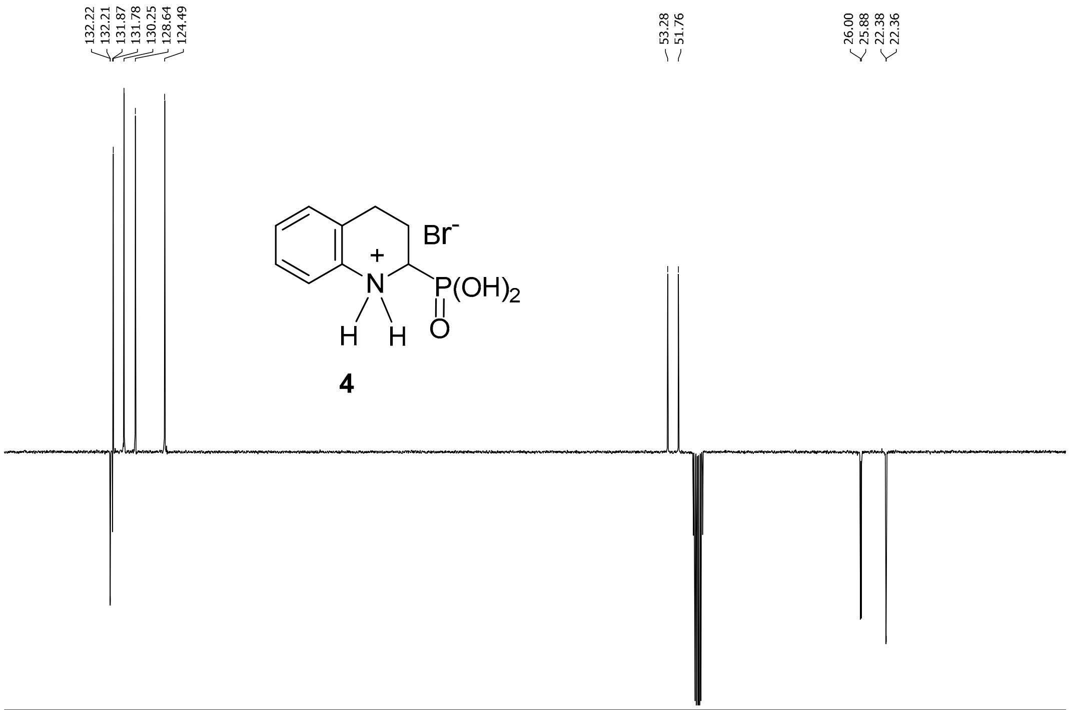

Figure S7. ^{13}C-NMR of **4** (100 MHz, CD_3OD).

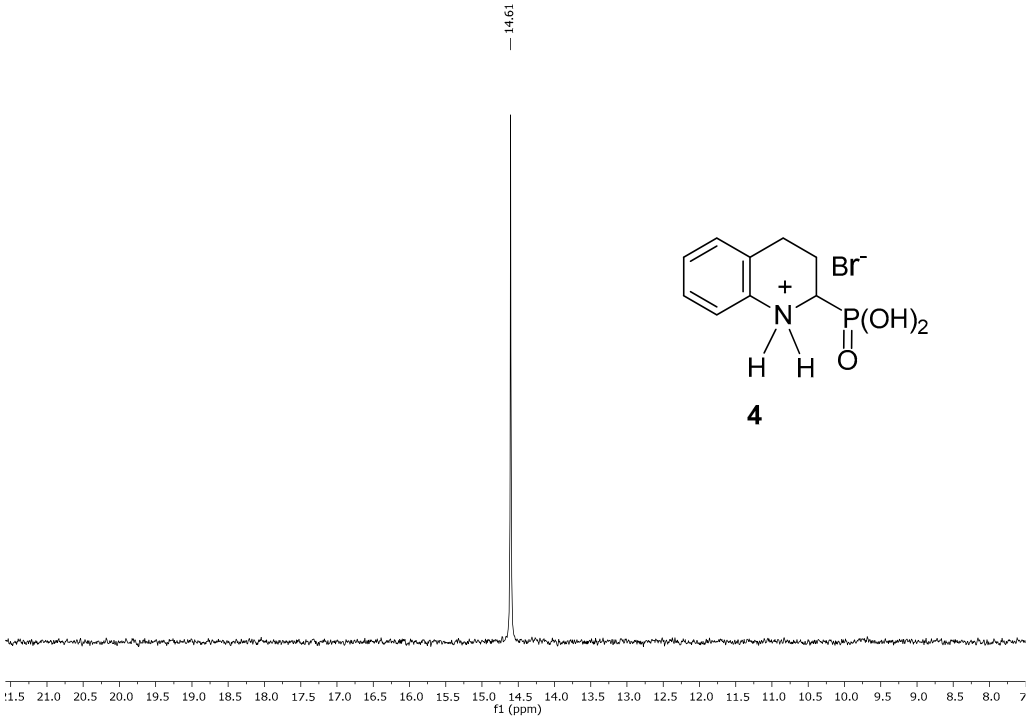

Figure S8. ^{31}P-NMR of **4** (162 MHz, CD_3OD).

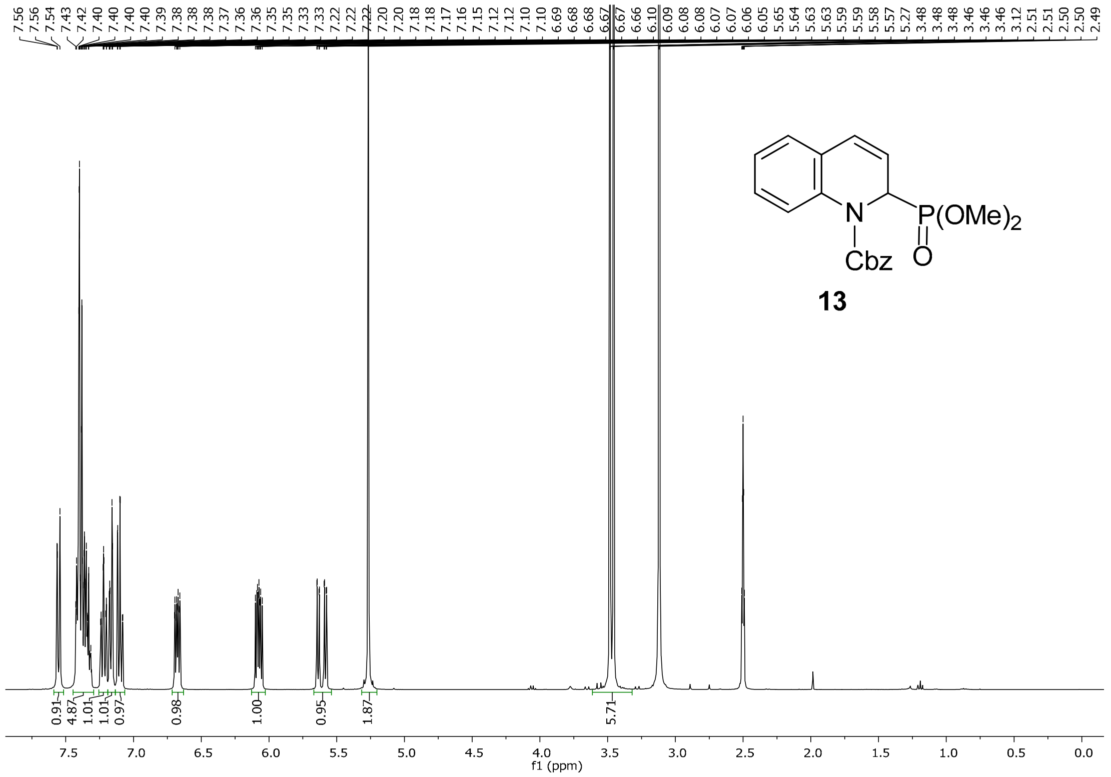

Figure S9. ^{1}H-NMR of **13** (400 MHz, DMSO-d_6, 80 °C).

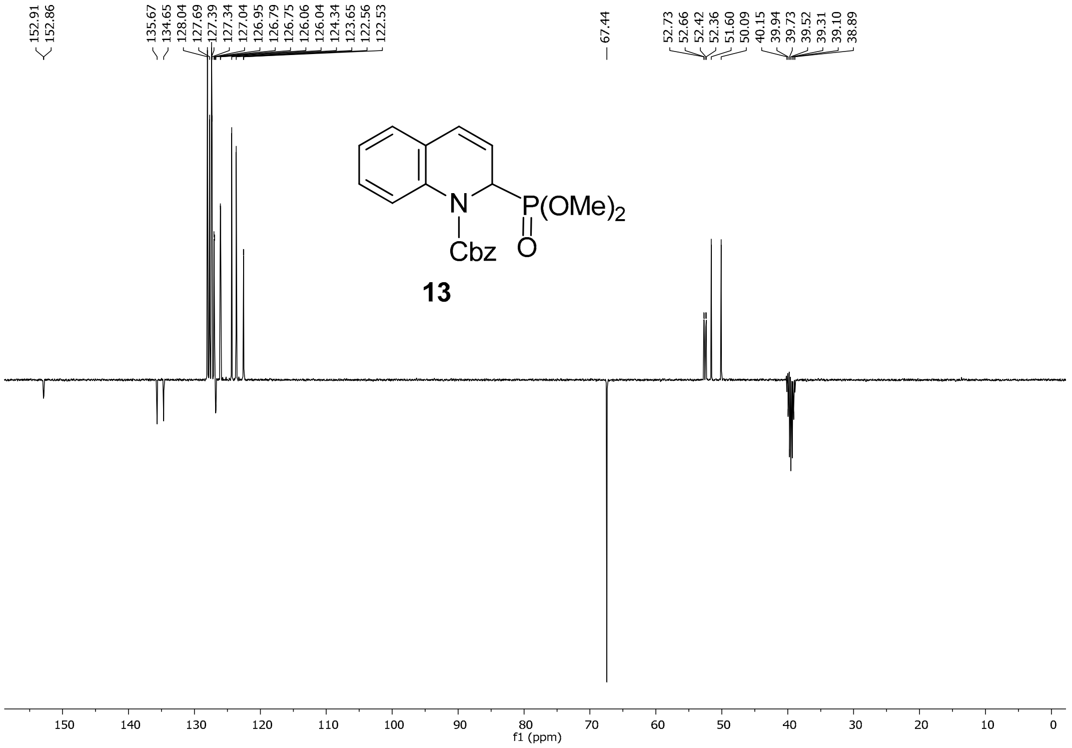

Figure S10. ^{13}C-NMR of **13** (100 MHz, DMSO-*d*$_6$, 80 °C).

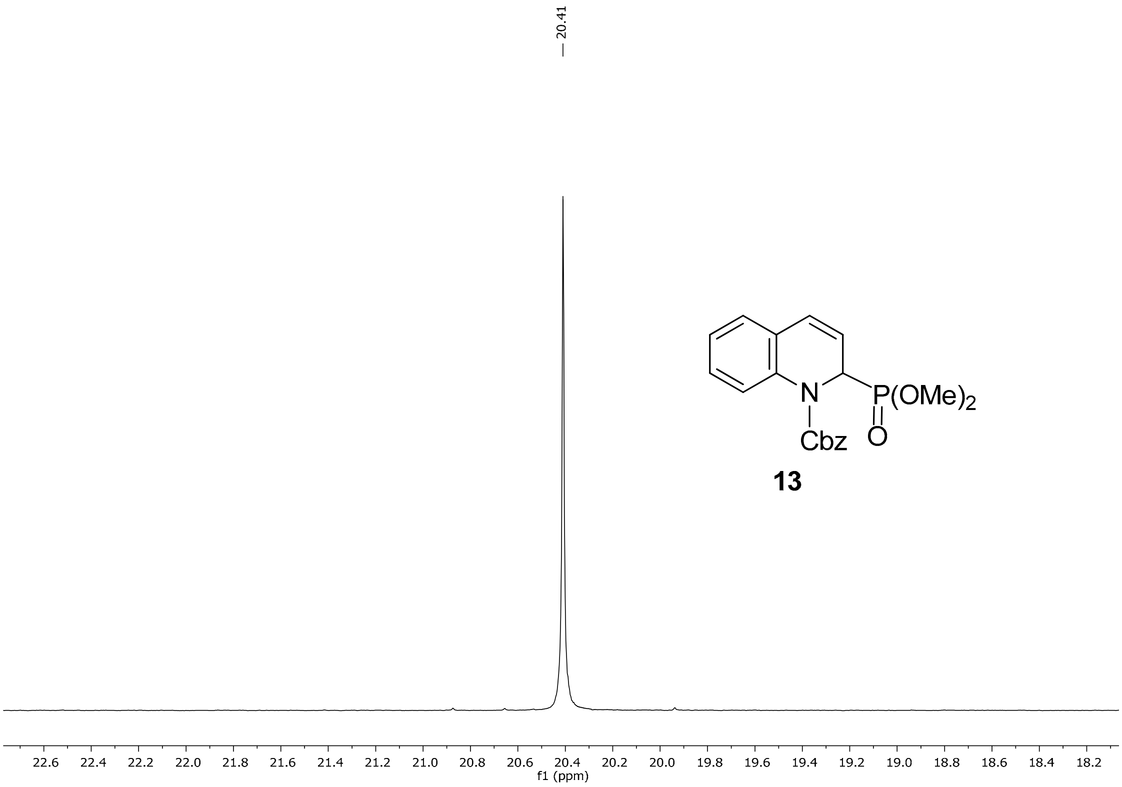

Figure S11. ^{31}P-NMR of **13** (162 MHz, DMSO-*d*$_6$, 80 °C).

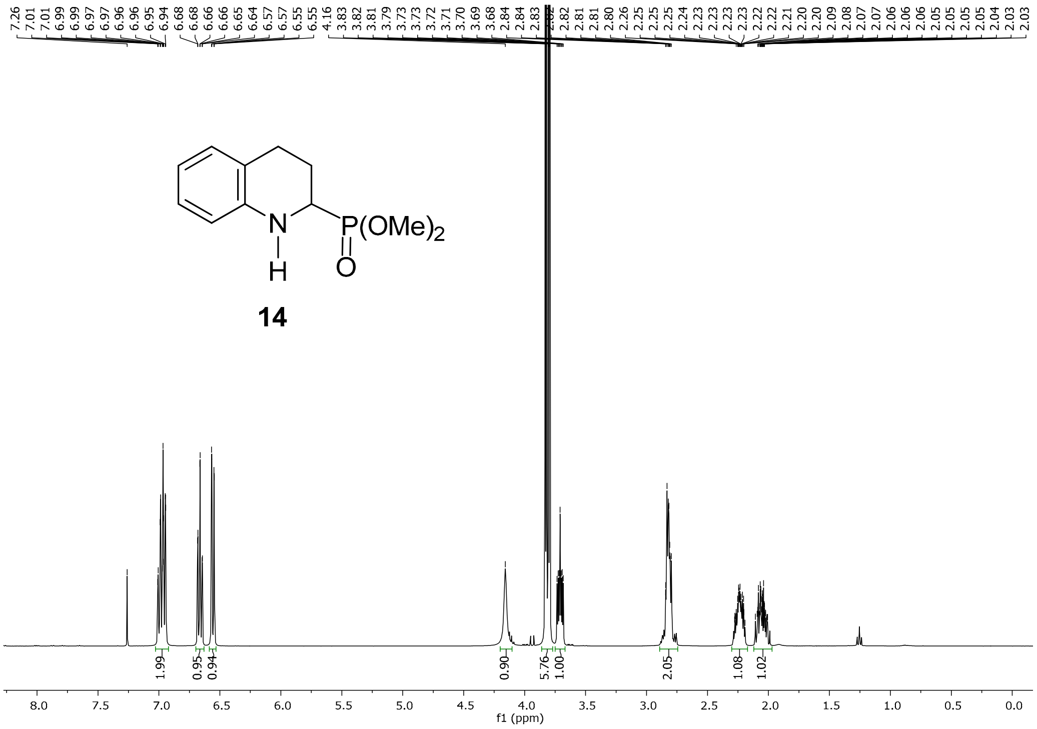

Figure S12. ^{1}H-NMR of **14** (400 MHz, $CDCl_3$).

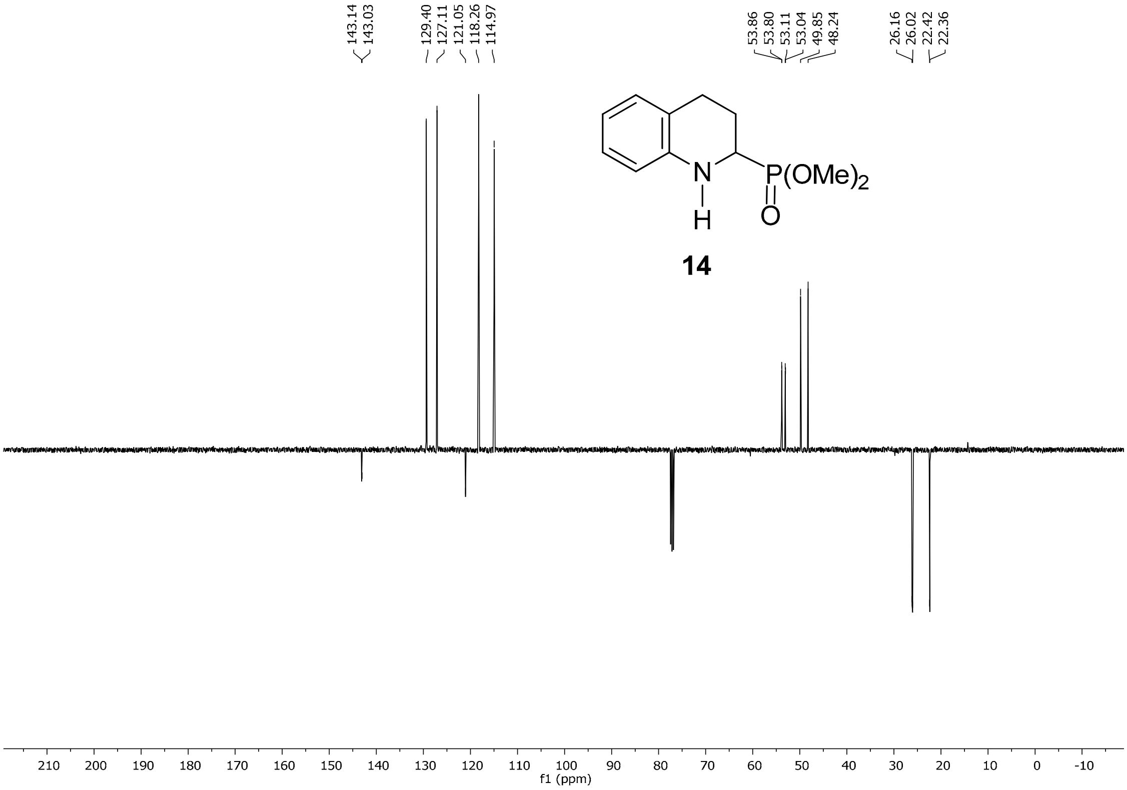

Figure S13. ^{13}C-NMR of **14** (100 MHz, $CDCl_3$).

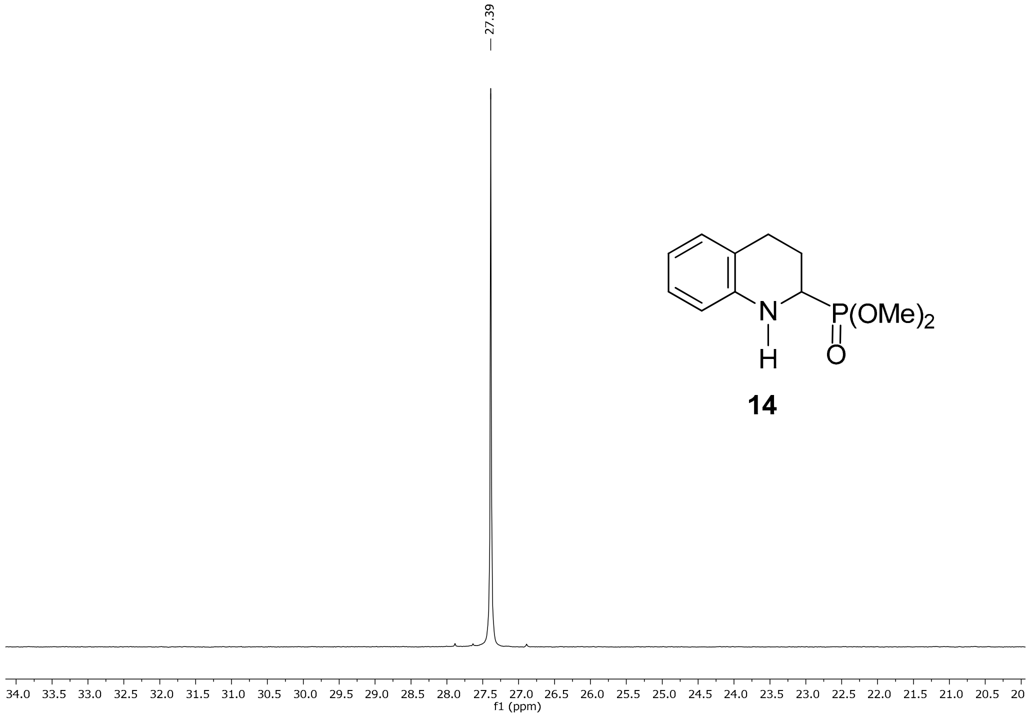

Figure S14. ^{31}P-NMR of **14** (162 MHz, $CDCl_3$).

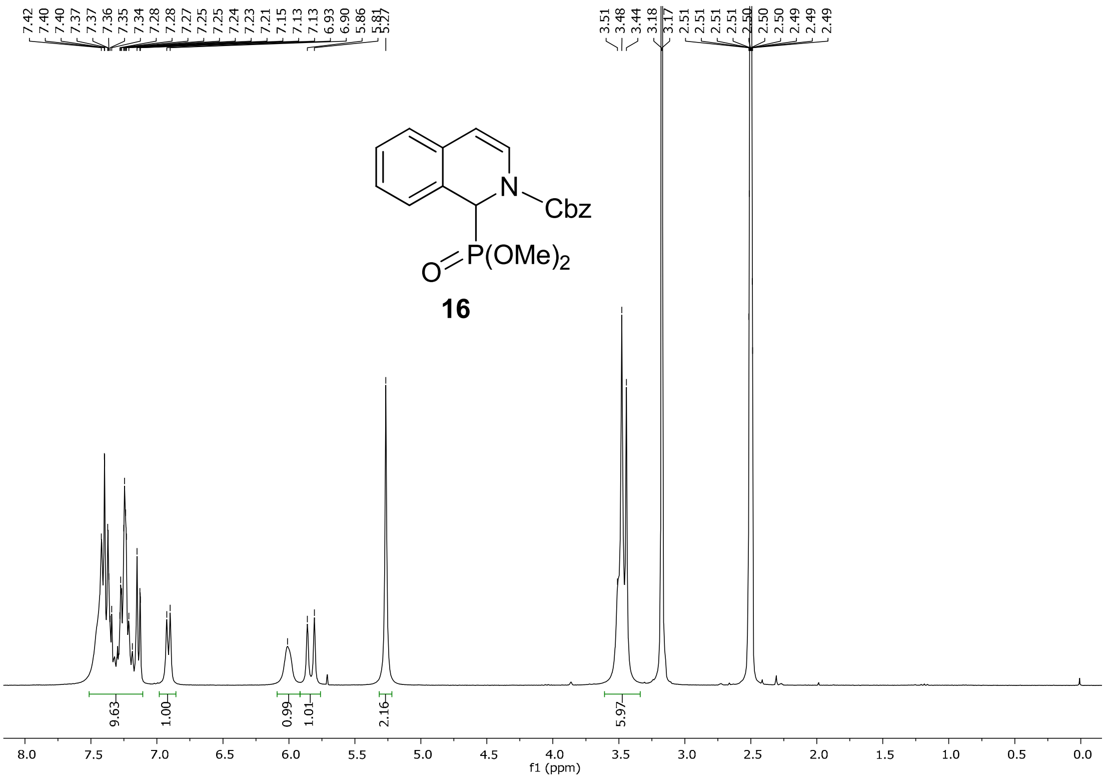

Figure S15. ^{1}H-NMR of **16** (300 MHz, DMSO-d_6, 50 °C).

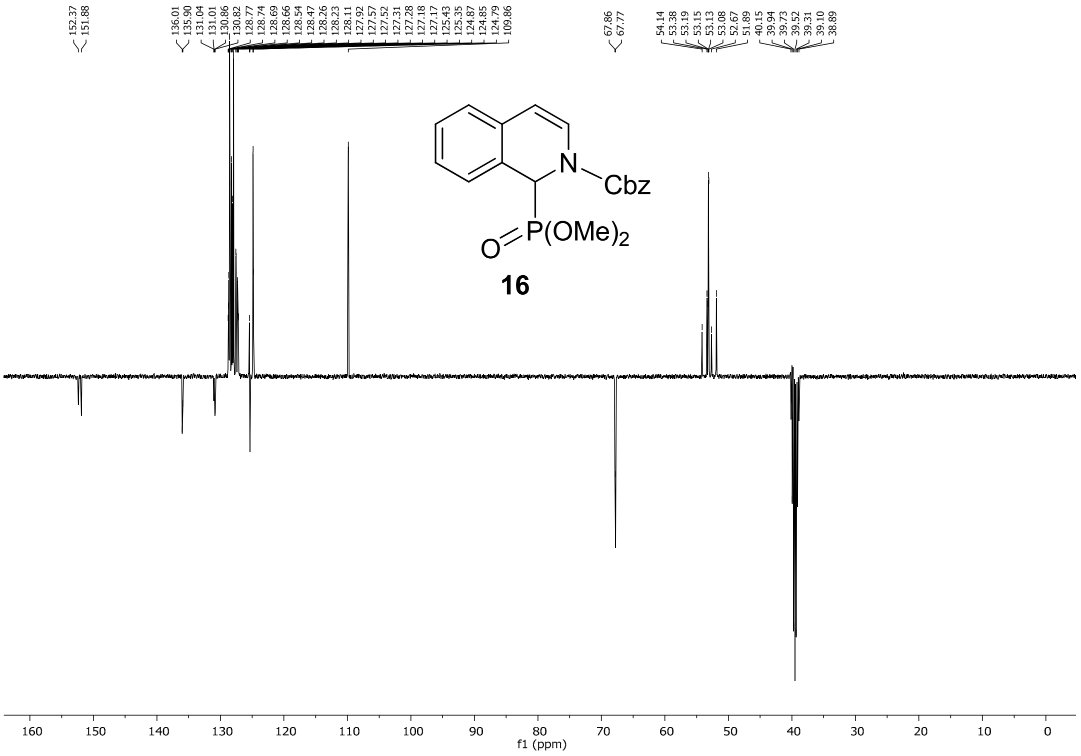

Figure S16. ^{13}C-NMR of **16** (100 MHz, DMSO-*d*$_6$).

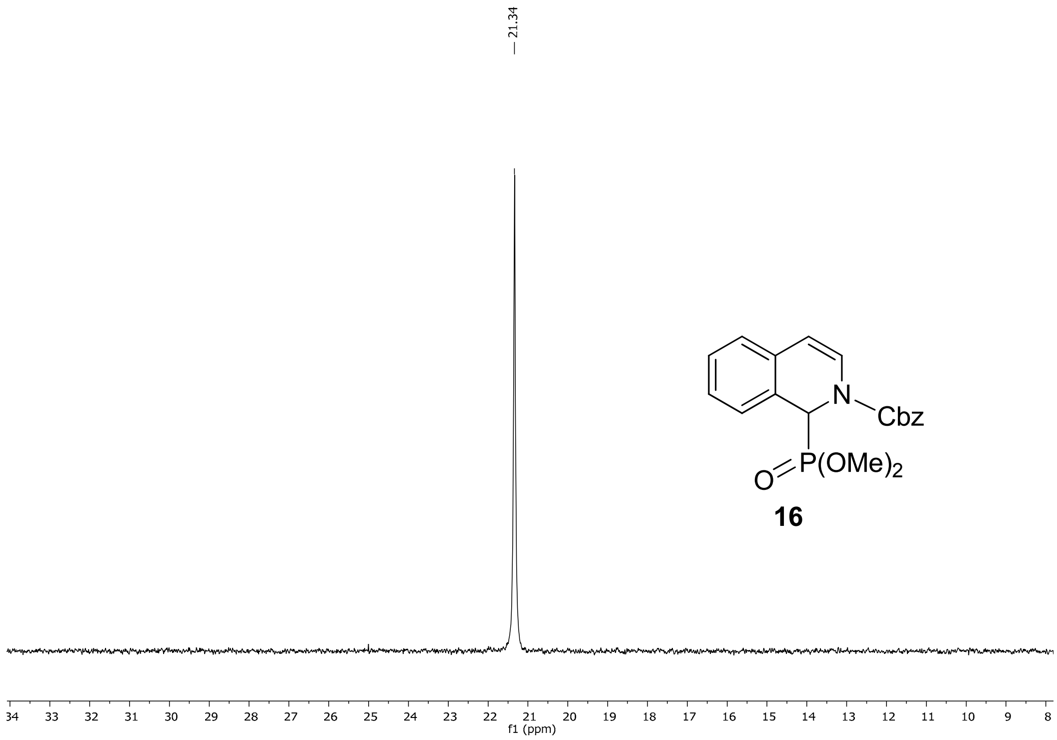

Figure S17. ^{31}P-NMR of **16** (122 MHz, DMSO-*d*$_6$, 50 °C).

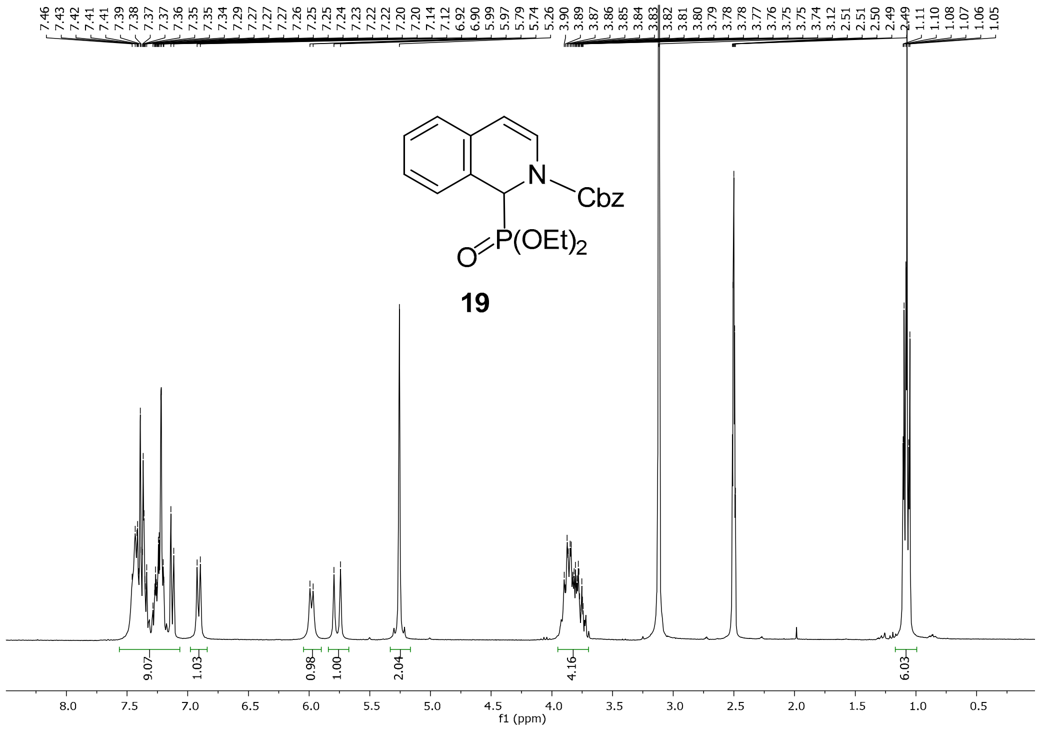

Figure S18. ^{1}H-NMR of **19** (300 MHz, DMSO-d_6, 70 °C).

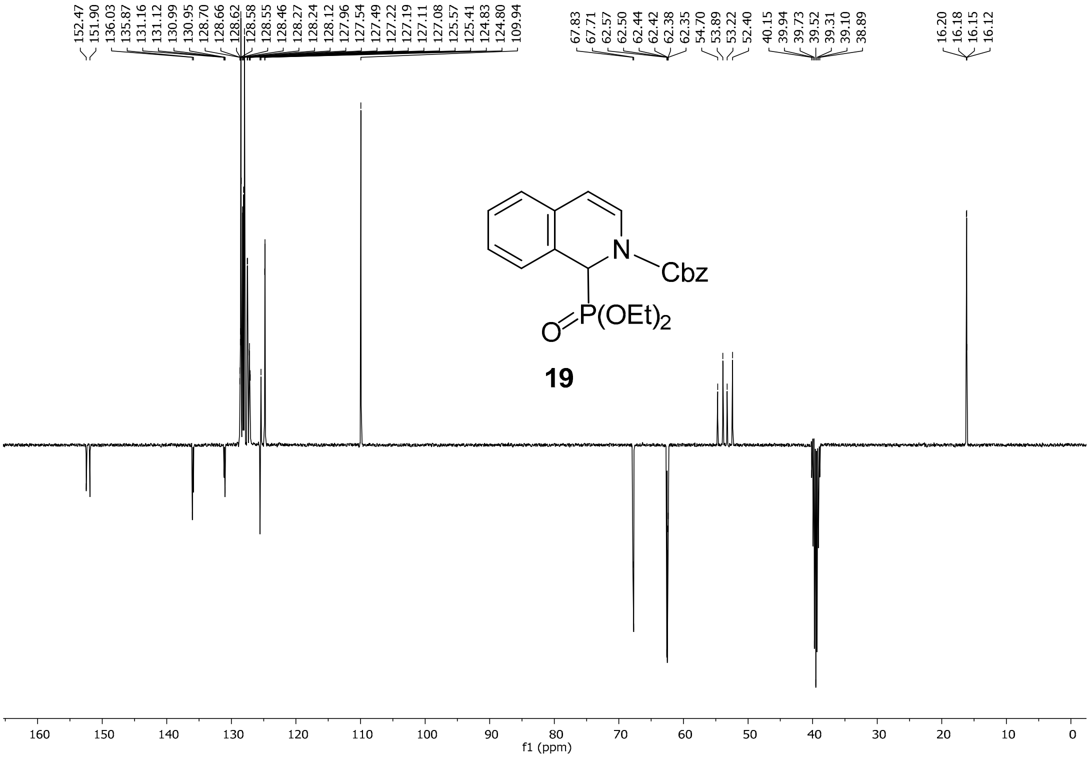

Figure S19. ^{13}C-NMR of **19** (100 MHz, DMSO-d_6).

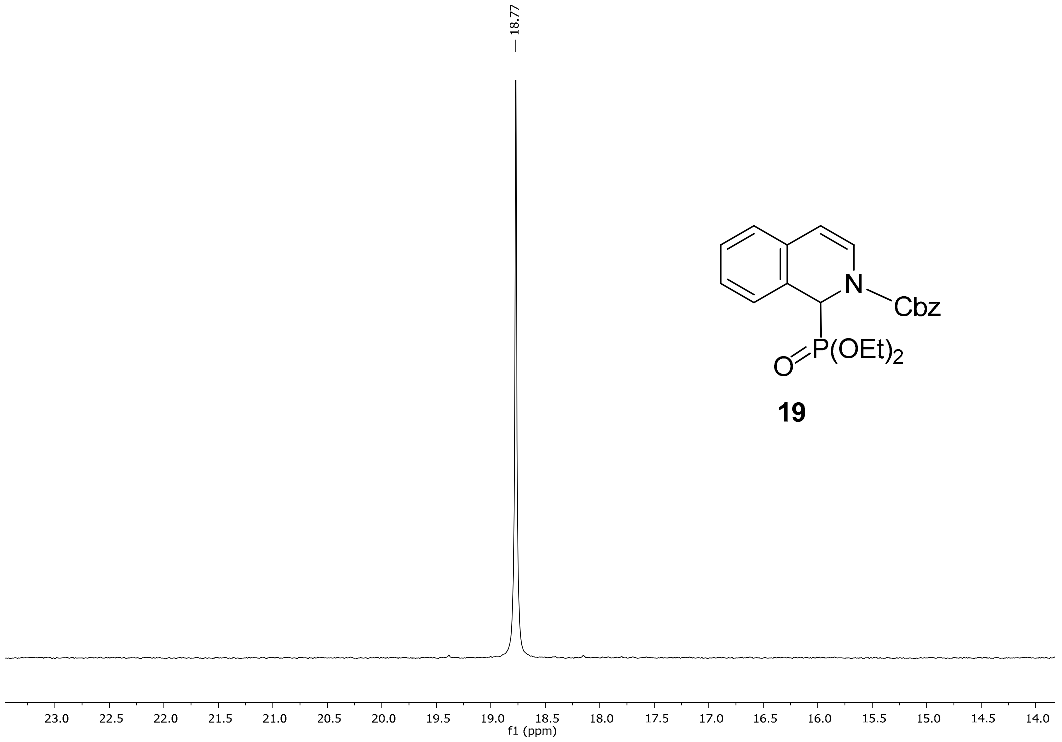

Figure S20. ^{31}P-NMR of **19** (122 MHz, DMSO-d_6, 70 °C).

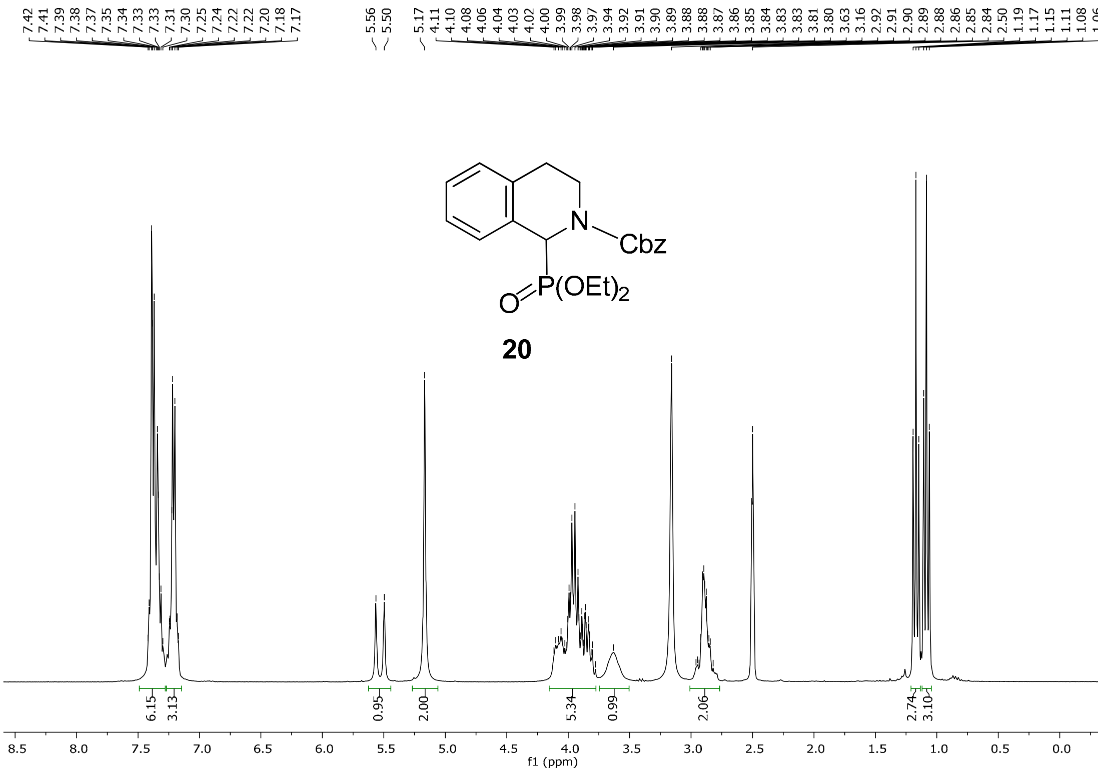

Figure S21. ^{1}H-NMR of **20** (300 MHz, DMSO-d_6, 70 °C).

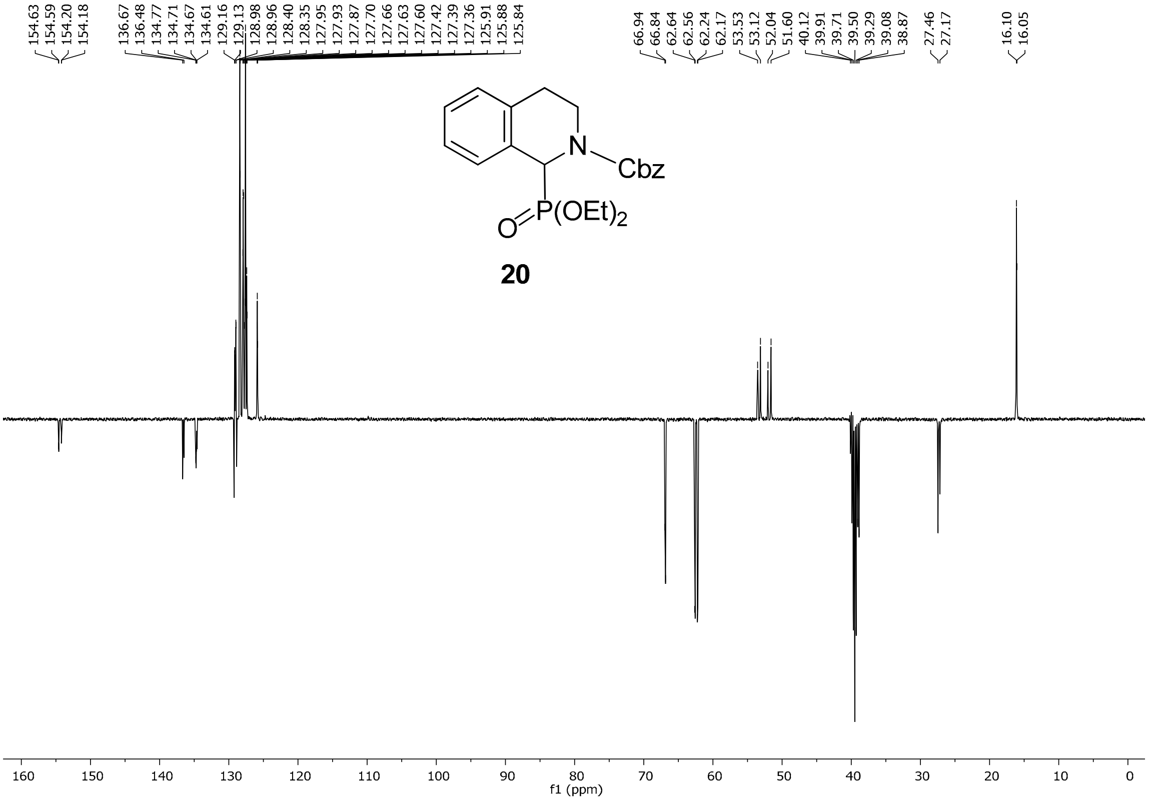

Figure S22. ^{13}C-NMR of **20** (100 MHz, DMSO-d_6).

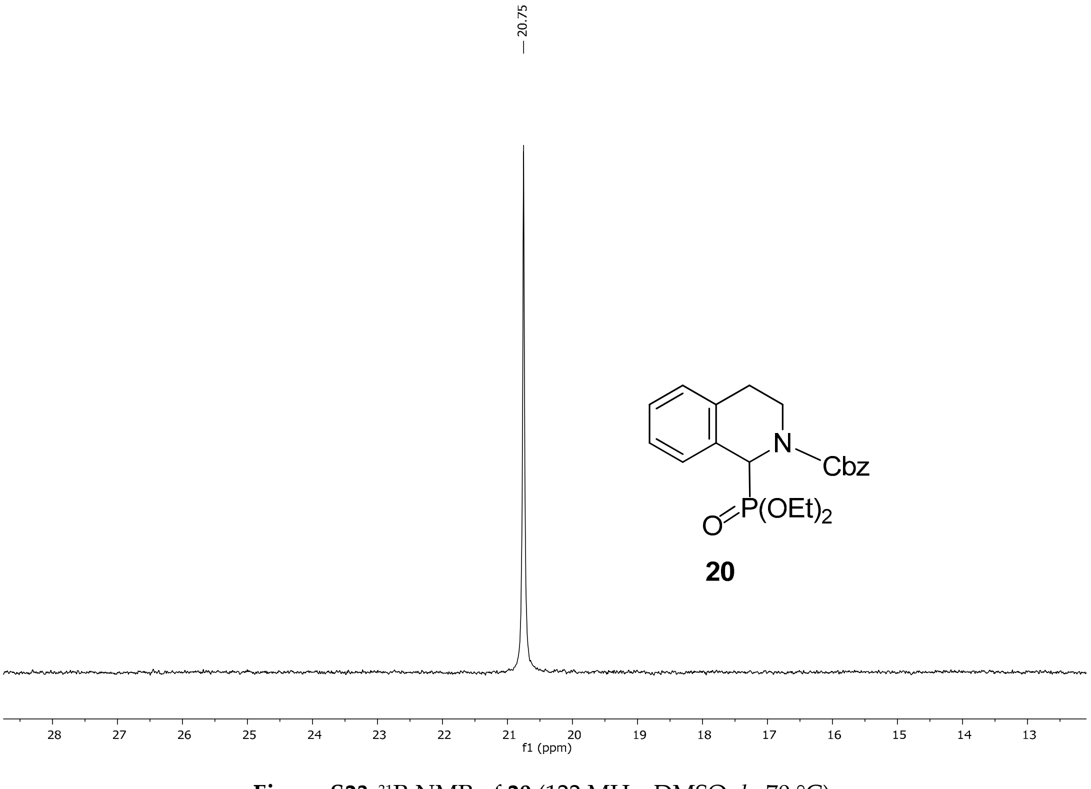

Figure S23. ^{31}P-NMR of **20** (122 MHz, DMSO-d_6, 70 °C).

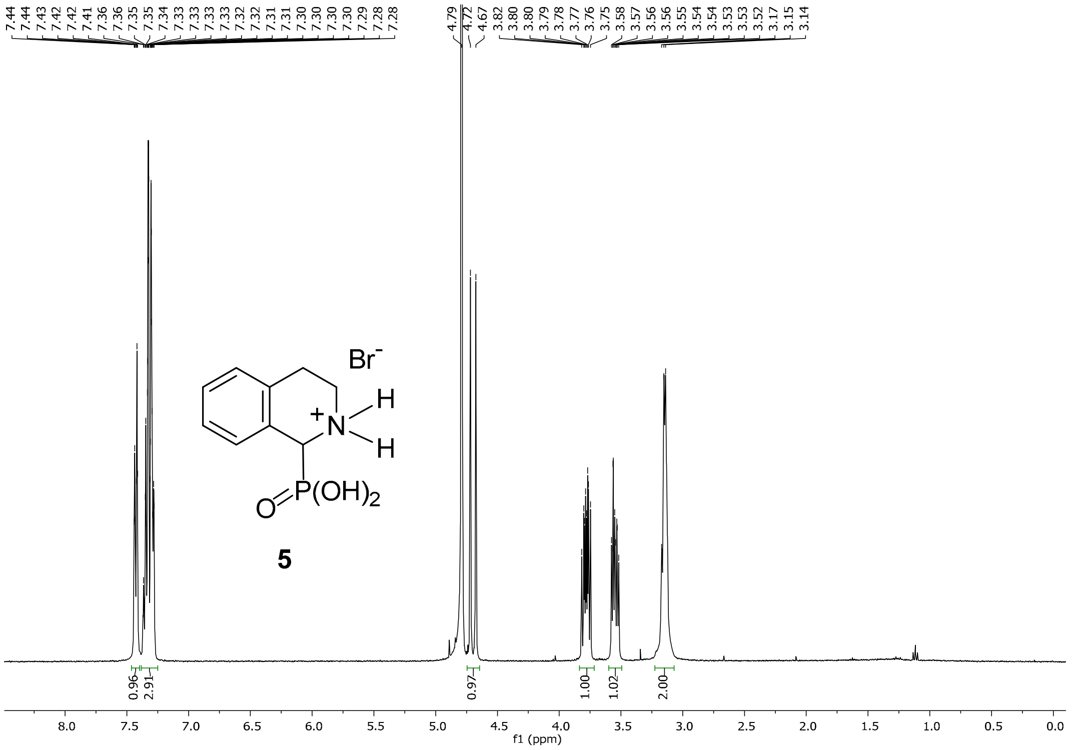

Figure S24. ^{1}H-NMR of **5** (400 MHz, D_2O).

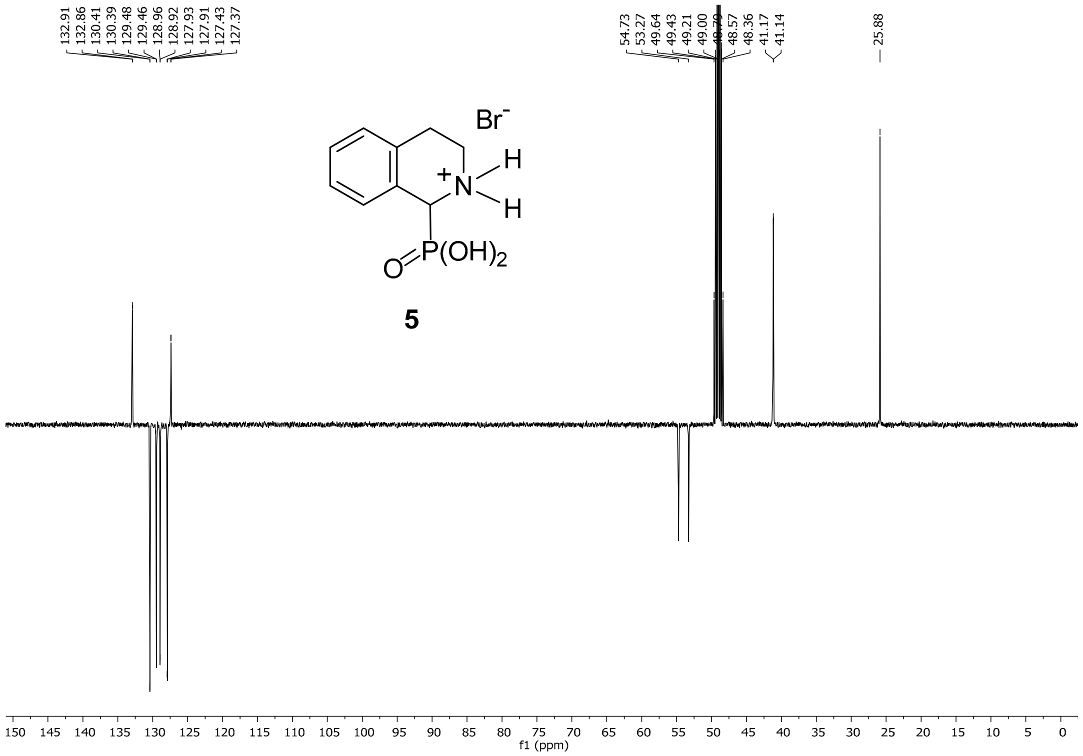

Figure S25. ^{13}C-NMR of **5** (100 MHz, CD_3OD).

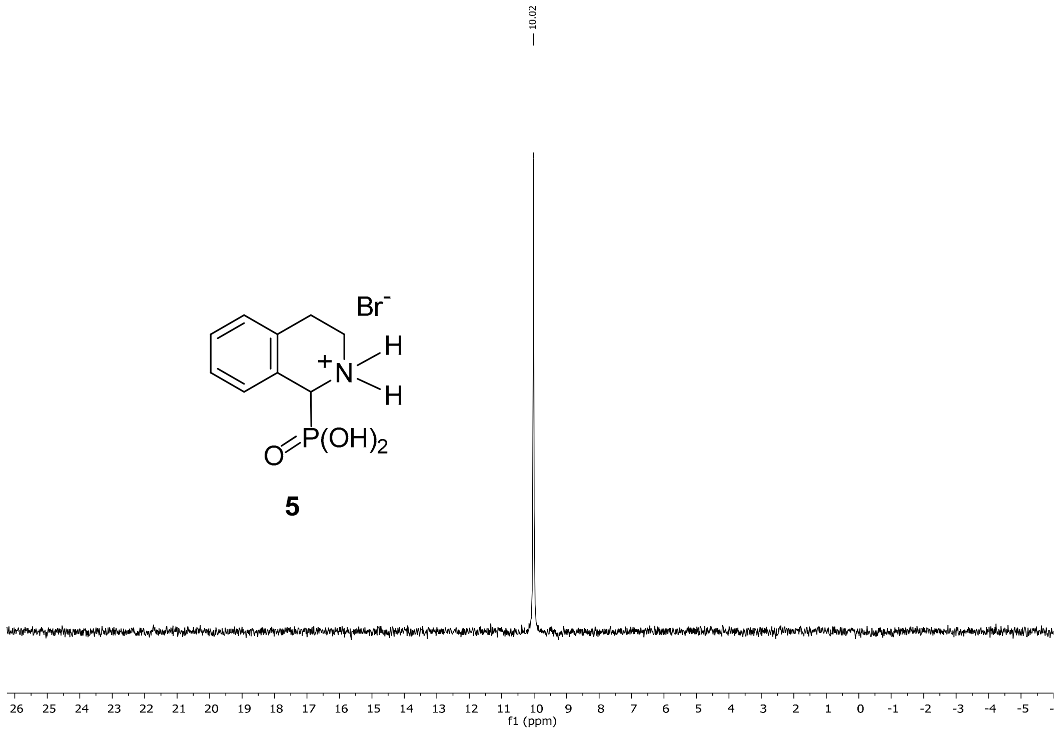

Figure S26. ^{31}P-NMR of **5** (162 MHz, D_2O).

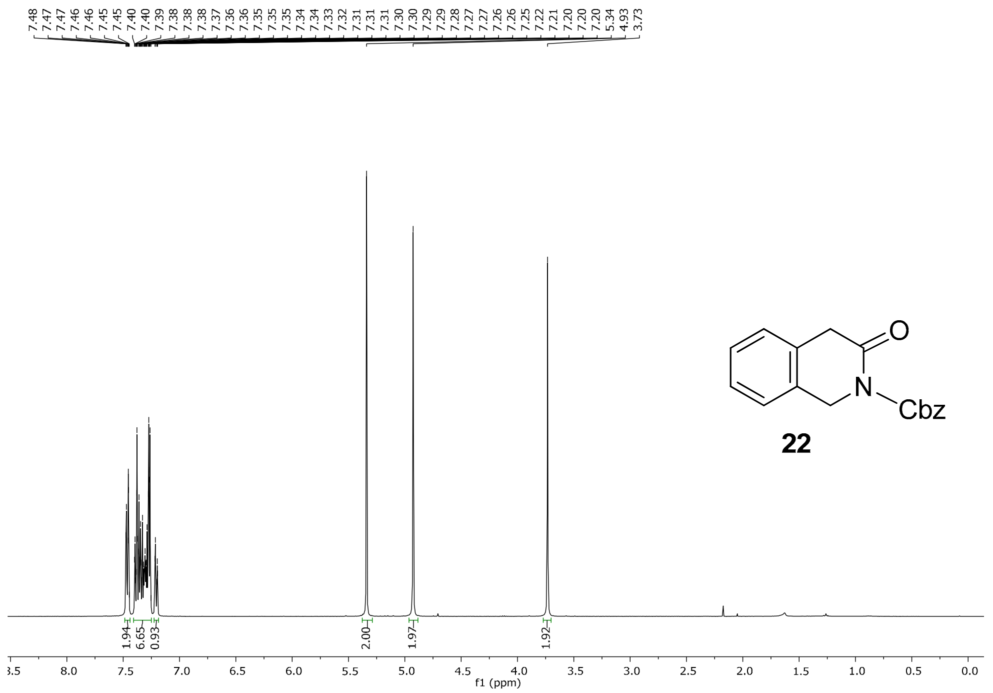

Figure S27. ^{1}H-NMR of **22** (400 MHz, $CDCl_3$).

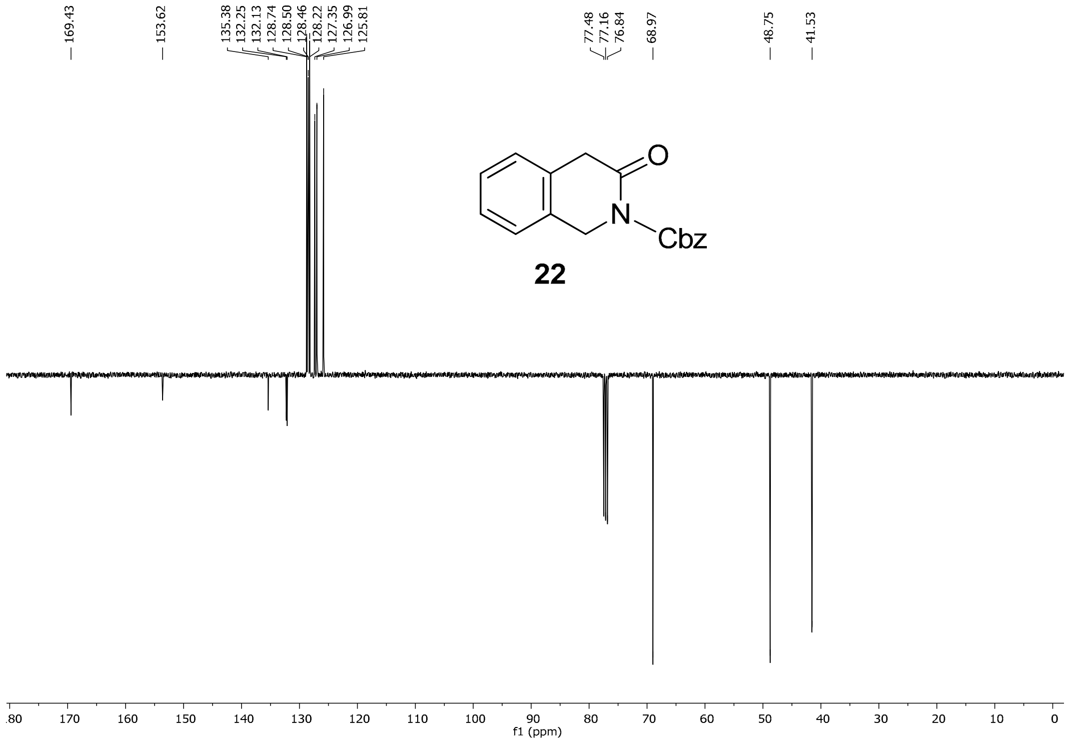

Figure S28. ^{13}C-NMR of **22** (100 MHz, C

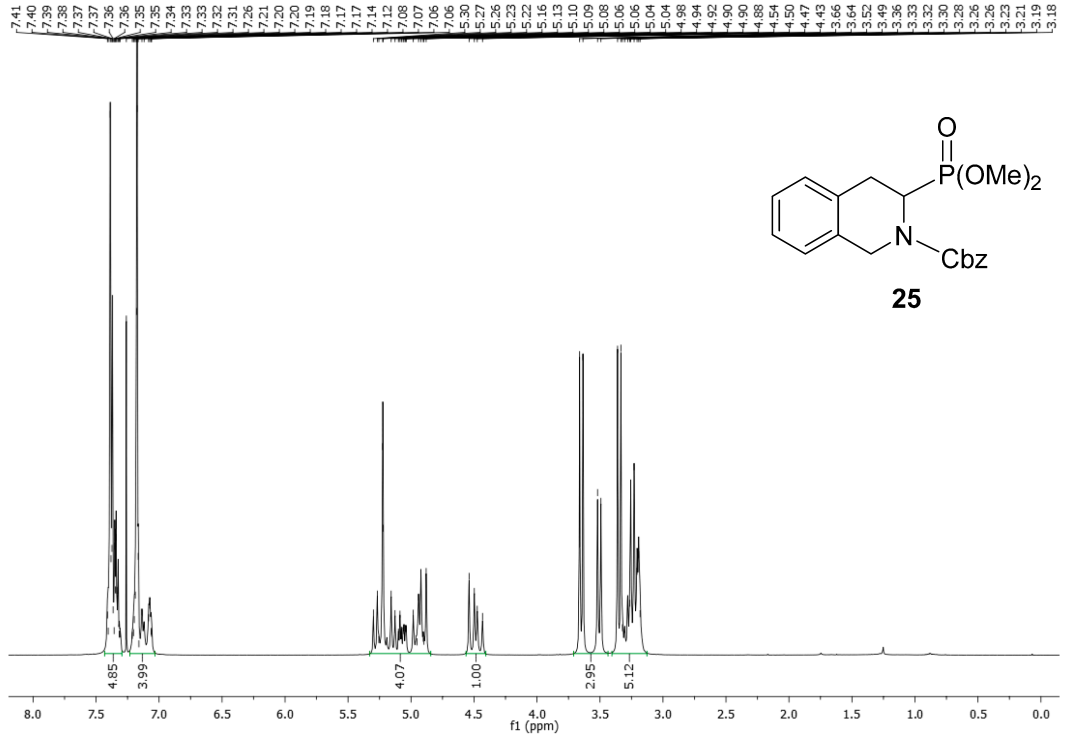

Figure S29. ^{1}H-NMR of **25** (400 MHz, $CDCl_3$).

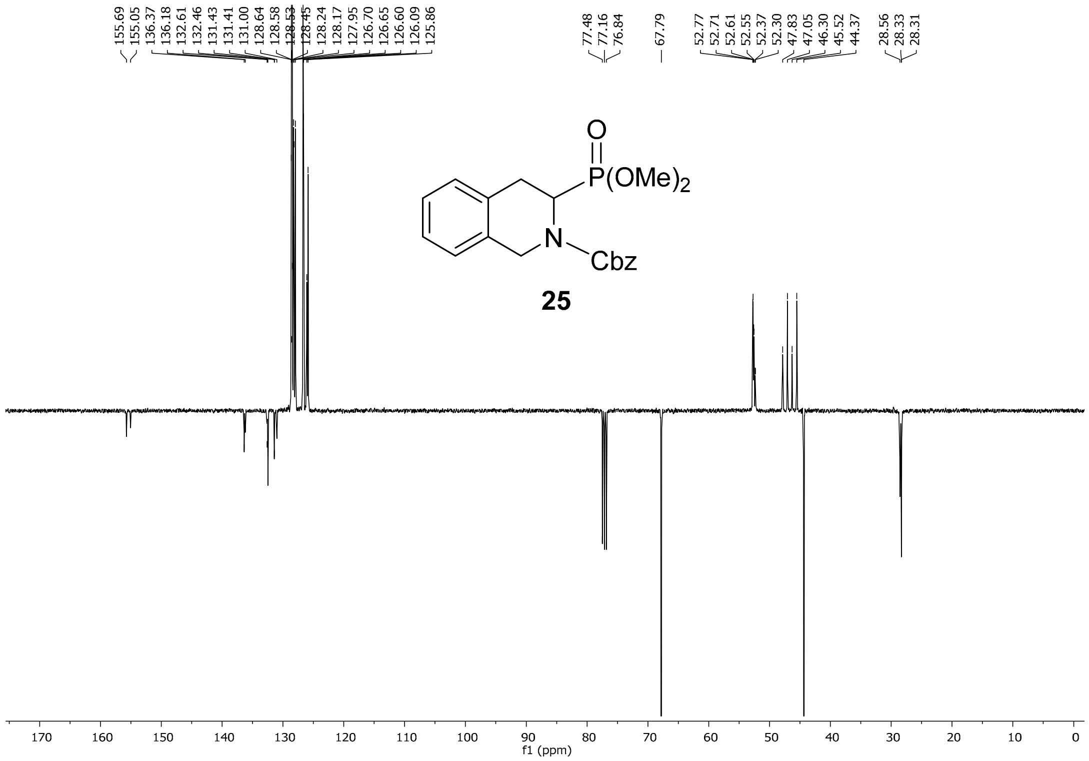

Figure S30. ^{13}C-NMR of **25** (100 MHz, $CDCl_3$).

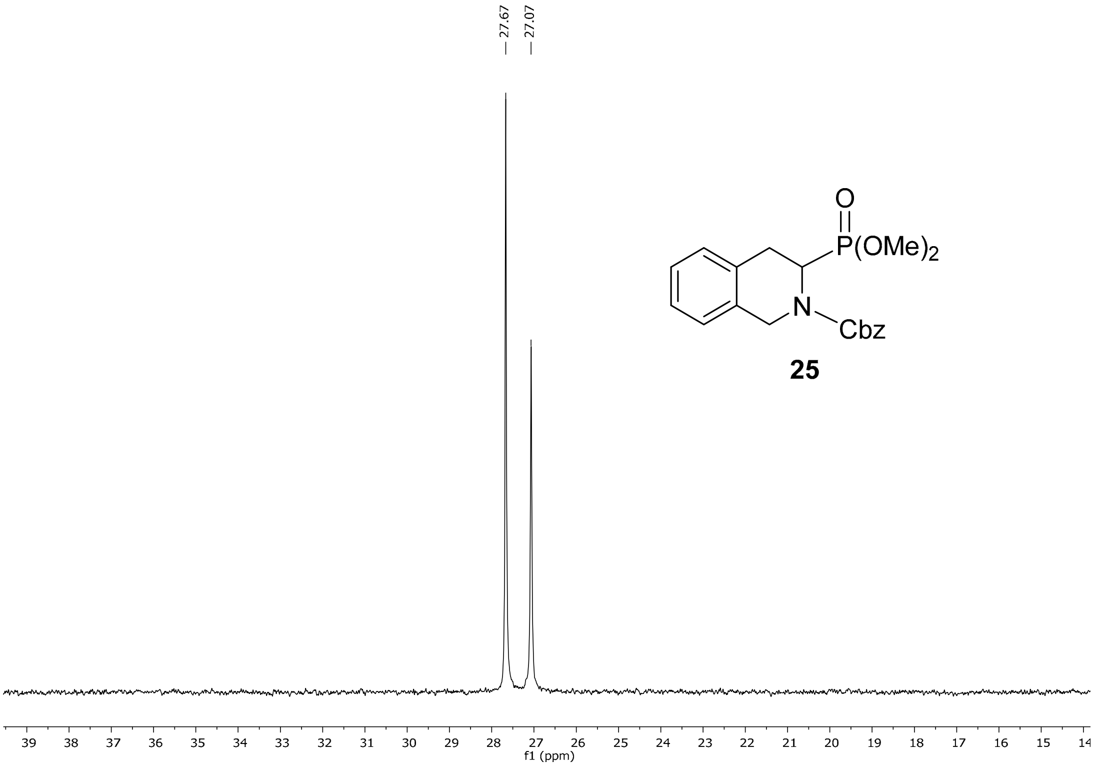

Figure S31. ^{31}P-NMR of **25** (162 MHz, $CDCl_3$).

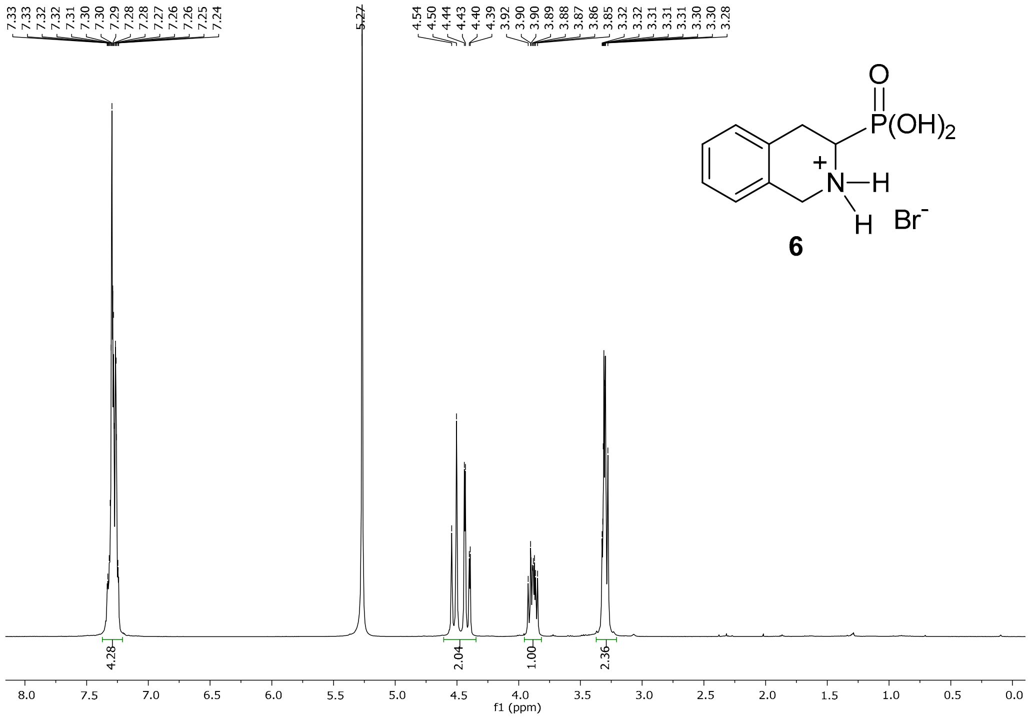

Figure S32. ^{1}H-NMR of **6** (400 MHz, CD_3OD).

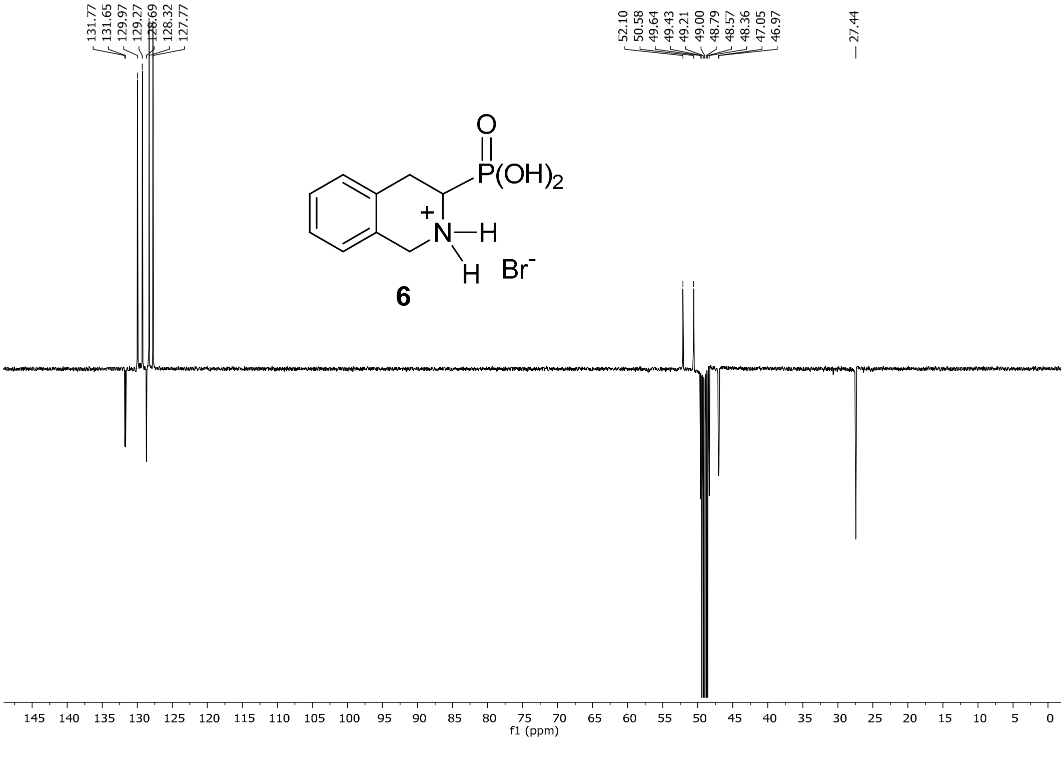

Figure S33. ^{13}C-NMR of **6** (100 MHz, CD_3OD).

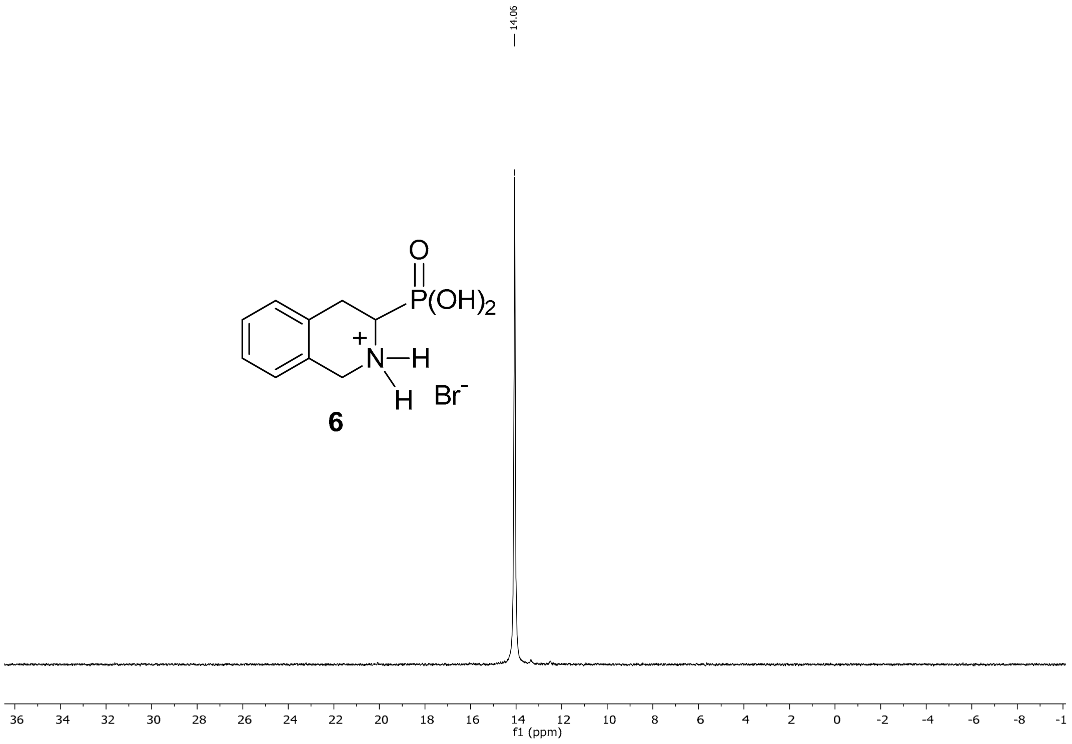

Figure S34. ^{31}P-NMR of **6** (162 MHz, CD_3OD).
